# Progressing towards the 2030 health-related SDGs in ASEAN: A systematic analysis

**DOI:** 10.1371/journal.pmed.1004551

**Published:** 2025-04-21

**Authors:** Yafei Si, Lei Guo, Shu Chen, Xinyu Zhang, Xiaochen Dai, Daniel Wang, Yunguo Liu, Bach Xuan Tran, Paul Michael Pronyk, Shenglan Tang

**Affiliations:** 1 Melbourne School of Population & Global Health, The University of Melbourne, Parkville, Victoria, Australia; 2 Global Health Research Center, Duke Kunshan University, Kunshan, China; 3 Duke Global Health Institute, Duke University, Durham, North Carolina, United States of America; 4 Department of Health Metrics Science, School of Medicine, Institute for Health Metrics and Evaluation, University of Washington, Seattle, Washington, United States of America; 5 School of Medicine, Duke University, Durham, North Carolina, United States of America; 6 Faculty of Public Health, VNU University of Medicine and Pharmacy, Vietnam National University, Hanoi (VNU-UMP), Hanoi, Vietnam; 7 SingHealth Duke-NUS Global Health Institute, Duke-NUS Medical School, Singapore, Singapore; 8 Duke-NUS Centre for Outbreak Preparedness, Duke-NUS Medical School, Singapore, Singapore; 9 Department of Population Health Science, Duke Medical School, Durham, North Carolina, United States of America; Harvard University, UNITED STATES OF AMERICA

## Abstract

**Background:**

The Sustainable Development Goals (SDGs) articulate an ambitious global agenda and set of targets to achieve by 2030. Among the health-related SDGs, many formidable challenges remain in settings like the Association of Southeast Asian Nations (ASEAN) which face wide-ranging social, economic and health inequalities. In advance of the 2030 horizon, charting the trajectory of the health SDGs is critical for informing policy and programmatic course corrections to advance health and well-being among ASEAN’s 10 member countries with its 667 million people.

**Methods and findings:**

We used estimates from the Global Burden of Disease (GBD) Study 2021 and surveillance data to identify 27 health-related SDG indicators. The indicators were classified into 7 thematic areas: (i) nutrition, (ii) maternal, child and reproductive health (MCH), (iii) infectious diseases, (iv) non-communicable diseases (NCDs), (v) environmental health, (vi) universal health coverage (UHC), and (vii) road injuries. We developed an attainment index ranging from 0 to 100 for each SDG indicator by referencing the SDG targets and projected their progress to 2030.

We find an overall positive progress towards the health-related SDG targets in ASEAN from 1990 to 2030. At the aggregate level by 2030, 2 member countries, Singapore and Brunei, are projected to achieve their targets (attainment score ≥ 90). At a wider regional level, ASEAN is projected to make substantial progress in nutrition, MCH, and UHC, with a majority of countries projected to come close to or achieve their targets. However, progress is projected to be slower in the areas of reducing the incidence of infectious disease (i.e., HIV and AIDs, hepatitis B, TB, and neglected tropical diseases), NCD-related mortality and its risk factors (i.e., harmful alcohol use and smoking), environment-related mortality and its risk factors (i.e., unsafe water and poor hygiene, and air pollution), and road injuries. Substantial disparities are identified in the region, with Singapore, Brunei, Malaysia and Thailand generally performing better than elsewhere.

A limitation of our study was its reliance on historical trends which may not fully capture future political, social, or technological changes.

**Conclusions:**

As a regional bloc, ASEAN faces persistent challenges in achieving health-related SDG targets by 2030, with unequal progress between countries. Moreover, epidemiological transitions and worsening environmental threats further compound potential gains. At the country level, efforts to enhance health system financing, quality and equity will need to be coupled with wider approaches that address structural drivers of disease. Furthermore, coordinated regional efforts will be essential to effectively respond to emerging threats posed by pollution and environmental risks.

## Summary

**Why was this study done?:**

- The Association of Southeast Asian Nations (ASEAN) aim to meet health related Sustainable Development Goals (SDGs) by 2030, but are facing significant challenges due to uneven economic and social development
- Understanding each country’s progress can help identify gaps, inform policies, and guide future actions to improve health in the region.

**What did the researchers do and find?:**

- We analyzed data from 1990 to 2021 to measure ASEAN’s progress toward health targets in nutrition, maternal and child health, infectious and non-communicable chronic diseases, environment health, universal health coverage, and road injuries.
- Overall, countries improved in nutrition, maternal and child health, and universal health coverage, but they struggled to reduce infectious diseases, non-communicable chronic diseases, environmental risks, and road injuries.
- Singapore and Brunei are likely to achieve most targets by 2030, whereas countries like Laos, Philippines, and Cambodia face significant challenges.

**What do these findings mean?:**

- ASEAN countries should develop and implement robust strategies to address these health issues in order to improve effectively the health of their populations.
- Successful experiences from high-performing countries should be shared regionally to help those lagging behind.
- A limitation of our study was its reliance on historical trends which may not fully capture future political, social, or technological changes.

## 1. Introduction

The Millennium Development Goals (MDG) framework (2000–2015) was a global initiative developed by the United Nations, aiming to address major global challenges such as poverty, health, education, and gender equality [1,2]. After 2015, the Sustainable Development Goals (SDGs) have been seen as a global development agenda through 2030 to build upon the MDGs with a broader and more integrated framework that emphasized sustainability, inequality, and long-term development [3]. While the MDGs had a focus on reducing poverty and preventable deaths from priority conditions with a focus on the poorest [1], the SDGs outline a global agenda that is relevant for all countries. At its core, the health-related SDGs aim to secure an improvement in global health translating to healthier populations, reduced mortality rates, and increased life expectancy [2]. Among its 17 goals, SDG 3 (Good health and well-being) explicitly addresses health, with other goals advancing health-related gains directly or indirectly [4]. Since its launch, a range of global initiatives have been developed to track global progress toward health-related SDGs including the Atlas of the SDGs, Monitoring Health for the SDGs, and the Global Burden of Disease (GBD) Study [5–8].

The Association of Southeast Asian Nations (ASEAN) is a political and economic union of 10 member countries which include Brunei, Myanmar, Cambodia, Indonesia, Laos, Malaysia, Philippines, Singapore, Thailand and Vietnam [9]. ASEAN aims to keep stability via economic growth, social progress, and cultural development [10]. Indonesia, Philippines and Vietnam contain close to three-quarters of ASEAN’s combined population of over 667 million people. As a regional bloc, ASEAN represents the world’s 5th-largest economy and is projected to become the 4th-largest by 2030 [11], despite major disparities in economic development within the region. At the extremes, the per capita gross domestic product (GDPpc) in Singapore is 88,428 USD relative to 1,149 USD Myanmar (2022) [12]. Sustainability has recently emerged as a regional priority. ASEAN has explicitly committed to sustainability in its 2015 charter [13] and the AESAN Community Vision 2025 emphasizes a regional focus on environmental protection for now and the future, adapting and responding to climate change, green technology and development [14].

ASEAN made significant progress towards achieving the MDG targets by 2015, despite regional disparities. For example, ASEAN made significant strides in reducing poverty rates [15]. Vietnam, Cambodia, and Indonesia substantially reduced the proportion of people living in extreme poverty and decreased the proportion of stunting among children under 5 year old to 24.5%, 32.4%, and 27.5% in 2016 (versus 4.4% in Singapore) [16]. Indonesia achieved a 94.9% participation rate in pre-primary organized learning in 2016 [17]. Improved access to healthcare services, immunization programs, skilled birth attendance, and family planning services contributed to a significant reduction in child mortality rates and maternal mortality rates in the region [18–20]. In 2016, under-5 child mortality was 30.5 per 1,000 live births in ASEAN, and maternal mortality was 202.8 deaths per 100,000 live births [18]. In addition, increased awareness, more prevention programs, and improved access to treatment contributed to a decline in malaria prevalence [19], although Indonesia and Myanmar are still the high burden countries. From 2010 to 2015, AIDS-related deaths and new HIV infections have shown a declining trend in almost all high-burden countries [20].

Despite the above-mentioned progress, ASEAN clearly faces a range of structural challenges that threaten progress towards achieving the SDGs. Inequalities in socio-economic development, healthcare infrastructure and political stability are pervasive [21,22]. Consequently, progress in maternal and under-5 mortality rate is uneven across countries and within populations, despite the region overall achieved a 78% reduction in maternal mortality and a 76% reduction in under-5 mortality [23]. Universal health coverage and financial protection remain a challenge, as the region has the highest out-of-pocket health expenditure among all WHO regions [24]. Neglected tropical diseases [25] and hunger [26] remain prevalent. Over 70% of global child tuberculosis mortality occurs in ASEAN [27]. Specifically, the incidence of TB in Cambodia, Myanmar, and Laos was as high as 326, 201, 154 per 100,000 population in 2015 (versus 33 in Singapore) [28]. A rapid rise in motor vehicle use alongside lower quality road networks have escalated road traffic injuries [29–31]. Especially, 28, 25, and 20 deaths happened among 100,000 population in Thailand, Malaysia, and Cambodia in 2015 (versus 3 in Singapore) [32]. Furthermore, challenges related to deforestation, pollution, and natural resource management create new drivers of communicable and non-communicable disease[9]. Finally, the COVID pandemic has substantially contributed towards excess deaths [33–36], declines in life expectancy [37], and rising mental health problems [38].

We conducted a review that estimates progress towards the health-related SDGs in ASEAN between 1990 and 2030. The objectives of this work are to profile advances towards direct and indirect health-related targets at a regional level, highlight intraregional differences, and inform policy and programmatic course corrections in the lead up to 2030 [39].

## 2. Methods

### 2.1. Data sources

We used data from the Global Burden of Disease (GBD) Study 2021 including (1) estimates of morbidity, mortality, and prevalence of risk factors and their attributed morbidity and mortality from 1990–2021 at the national level; and (2) estimates of health-related SDG monitoring indicators from 1990 to 2021. Details of the GBD Study 2021 and its methodology have been reported elsewhere [40–43]. In addition, we also used complementary data from World Bank and World Health Organization (WHO) to account in indicators related to universal health coverage.

### 2.2. Selection of relevant SDG indicators

Following a previous similar study [44], we used four general inclusion criteria to select SDG indicators: (1) direct relevance to health improvement in ASEAN; (2) clear target value defined by the SDGs or policies/guidelines developed by international organizations (i.e., WHO); (3) availability of reliable data in ASEAN countries; and (4) potential the inform of health policy and programmatic interventions. Based on the criteria, we selected 27 key health-related SDG indicators from the GBD Study 2021 and publicly available data from World Bank and WHO datasets. The justification of excluding 9 indicators was provided in S1 Appendix. The 27 selected indicators were further categorized into 7 thematic areas including: (i) nutrition, (ii) maternal, child and reproductive health (MCH), (iii) infectious diseases, (iv) non-communicable diseases (NCDs), (v) environmental health, (vi) universal health coverage (UHC), and (vii) road injuries. The details are presented in Table 1.

**Table 1:** List of selected 27 health-related SDG indicators.

Topic	Outline	Indicator description	Target Source	Target description	Target year
Nutrition	2.2.1	Prevalence of stunting in children under 5 (%).	WHO	By 2025, a 40% reduction of the global number of children under five who are stunted. This target implies a relative reduction of 40% of the number of children stunted by the year 2025, compared to the baseline of 2010.	2025
	2.2.2a	Prevalence of wasting in children under 5 (%).	WHO	By 2025, reduce and maintain childhood wasting to less than 5%. This target implies that the global prevalence of childhood wasting of 8.6% estimated for 2010 should be reduced to less than 5% by 2025 and maintained below such levels.	2025
MCH	3.1.1	Maternal mortality ratio (maternal deaths per 100,000 livebirths) in women aged 10–54 years.	UN	By 2030, reduce the global maternal mortality ratio to less than 70 per 100,000 live births.	2030
	3.2.1	Under-5 mortality rate (probability of dying before the age of 5 per 1,000 livebirths).	UN	By 2030, end preventable deaths of newborns and children under 5 years of age, with all countries aiming to reduce neonatal mortality to at least as low as 12 per 1,000 live births and under-5 mortality to at least as low as 25 per 1,000 live births.	2030
	3.2.2	Neonatal mortality rate (probability of dying during the first 28 days of life per 1,000 livebirths).	UN	By 2030, end preventable deaths of newborns and children under 5 years of age, with all countries aiming to reduce neonatal mortality to at least as low as 12 per 1,000 live births and under-5 mortality to at least as low as 25 per 1,000 live births.	2030
Infectious Diseases	3.3.1	Age-standardized rate of new HIV infections (per 1,000 population).	UN	By 2030, end the epidemics of AIDS, tuberculosis, malaria and neglected tropical diseases and combat hepatitis, water-borne diseases and other communicable diseases.	2030
	3.3.2	Age-standardized rate of tuberculosis cases (per 100,000 population).	WHO	80% reduction in the TB incidence rate (new and relapse cases per 100,000 population per year) by 2030, compared with 2015.	2030
	3.3.3	Age-standardized rate of malaria cases (per 1,000 population).	WHO	Reduce malaria case incidence globally by 90% compared with 2015.	2030
	3.3.4	Age-standardized rate of hepatitis B incidence (per 100,000 population).	WHO	30% reduction in new cases of chronic viral hepatitis B and C infections by 2020, 90% reduction by 2030.	2030
	3.3.5	Age-standardized prevalence of the sum of 15 neglected tropical diseases (NTDs) (%).	WHO	90% reduction in people requiring interventions against neglected tropical diseases by 2030.	2030
NCD	3.4.1	Age-standardized death rate due to cardiovascular disease, cancer, diabetes, and chronic respiratory disease in populations aged 30–70 (per 100,000 population).	UN	By 2030, reduce by one third premature mortality from non-communicable diseases through prevention and treatment and promote mental health and well-being.	2030
	3.4.2	Age-standardized death rate due to self-harm (per 100,000 population).	UN	By 2030, reduce by one third premature mortality from non-communicable diseases through prevention and treatment and promote mental health and well-being.	2030
	3.5.2	Risk-weighted prevalence of alcohol consumption, as measured by the summary exposure value (SEV) for alcohol use (%).	WHO	At least a 10% relative reduction in the harmful use of alcohol, as appropriate, within the national context (against baseline in 2010).	2030
	3.a.1	Age-standardized prevalence of current smoking in populations aged 10 and older (%).	WHO	A 30% relative reduction in prevalence of current tobacco use in persons aged 15+ years. (baseline of 2010).	2030
Environmental Health	3.9.1	Age-standardized death rate attributable to household air pollution and ambient air pollution (per 100,000 population).	UN	By 2030, substantially reduce the number of deaths and illnesses from hazardous chemicals and air, water and soil pollution and contamination.	2030
	3.9.2	Age-standardized death rate attributable to unsafe water, sanitation, and hygiene (WaSH) (per 100,000 population).	UN	By 2030, substantially reduce the number of deaths and illnesses from hazardous chemicals and air, water and soil pollution and contamination.	2030
	3.9.3	Age-standardized death rate due to unintentional poisonings (per 100,000 population).	UN	By 2030, substantially reduce the number of deaths and illnesses from hazardous chemicals and air, water and soil pollution and contamination.	2030
	6.1.1	Risk-weighted prevalence of populations using unsafe or unimproved water sources, as measured by the summary exposure value (SEV) for unsafe water (%).	UN	By 2030, achieve universal and equitable access to safe and affordable drinking water for all.	2030
	6.2.1a	Risk-weighted prevalence of populations using unsafe or unimproved sanitation, as measured by the summary exposure value (SEV) for unsafe sanitation (%).	UN	By 2030, achieve access to adequate and equitable sanitation and hygiene for all and end open defecation, paying special attention to the needs of women and girls and those in vulnerable situations.	2030
	6.2.1b	Risk-weighted prevalence of populations without access to a handwashing facility, as measured by the summary exposure value (SEV) for unsafe hygiene (%).	UN	By 2030, achieve access to adequate and equitable sanitation and hygiene for all and end open defecation, paying special attention to the needs of women and girls and those in vulnerable situations.	2030
	7.1.2	Risk-weighted prevalence of household air pollution, as measured by the summary exposure value (SEV) for household air pollution (%).	UN	By 2030, ensure universal access to affordable, reliable and modern energy services.	2030
	11.6.2	Population-weighted mean levels of fine particulate matter smaller than 2.5 microns in diameter (PM2.5).	WHO	An annual average concentration of 10 μg/m^3^ was chosen as the long-term guideline value for PM2.5.	2030
UHC	3.8.1	Coverage of essential health services, as defined by the UHC service coverage index of 9 tracer interventions and risk-standardized death rates or mortality-to-incidence ratios from 32 causes amenable to personal healthcare.	UN	Achieve universal health coverage, including financial risk protection, access to quality essential healthcare services and access to safe, effective, quality and affordable essential medicines and vaccines for all.	2030
	3.c.1a	Number of medical doctors (per 10,000 population).	WHO	Substantially increase health financing and the recruitment, development, training and retention of the health workforce in developing countries, especially in least developed countries and small island developing States.	2030
	3.c.1b	Number of nursing and midwifery personnel (per 10,000 population).	WHO	Substantially increase health financing and the recruitment, development, training and retention of the health workforce in developing countries, especially in least developed countries and small island developing States.	2030
	3.c.1c	Number of pharmacists (per 10,000 population).	WHO	Substantially increase health financing and the recruitment, development, training and retention of the health workforce in developing countries, especially in least developed countries and small island developing States.	2030
Injury	3.6.1	Age-standardized death rate due to road injuries (per 100,000 population).	UN	By 2020, halve the number of global deaths and injuries from road traffic accidents.	2020

Note: MCH, maternal and child health; NCD, non-communicable diseases; UHC, universal health coverage. Indicator 3.4.1 with specific age groups is the best fit the authors could find for the design of the study.

### 2.3. The measurement of SDG indicators

To make the progress in SDG indicators comparable, we transformed the values of the 27 indicators into an attainment index ranging from 0–100. Within each indicator, the worst value from 1990 to 2019 within the region was set as 0 and the target value was coded as 100. More details about the target values were described in S1 Appendix. The target year for most indicators is 2030, while for nutrition and road injuries the target years are 2025 and 2020, respectively set by WHO or UN [2,45]. We evaluated the attainment of each indicator by its target year and extrapolated the progress towards 2030. By calculating the geometric mean within each thematic area, we also generated an attainment index for each thematic area. An aggregated total score was calculated by considering all indicators and assigning equal weights to each SDG indicator [44]. Where values were less than one, we adjusted estimates to generate a valid geometric mean.

### 2.4. Projecting the attainment index of SDG indicators

We projected the absolute values for each indicator from 2020 to 2030 to rule out the short-term impact of the COVID-19 pandemic, as studies have documented a dip-down effect [46,47]. Specifically, (i) we defined a weight matrix *ω* as the trend of each indicator in each country; and (ii) we used the weighted mean annual rate of change to predict the data trend. This geometric rate of change assumes that a variable increases or decreases at the same rate during each year between the two time periods. In this case, we present the scaled mean scores without uncertainty intervals while GBD estimates with uncertainty intervals were presented in Table A1 in S1 Appendix. More details about the projection were described in S1 Appendix. We defined a threshold of 90 as the successful attainment of the health-related SDG targets. We plotted a choropleth map to visualize the attainment index by countries in 2030.

### 2.5. Annual change rate and regional disparity

To further compare the progress in each thematic area, we calculated the annual change rate of the attainment index from 1990 to 2030 by raising the change to an exponent of one divided by the number of years. The analysis was disaggregated at the country level. Inter-country disparities were explored and grouped by human development index (HDI) [48]. We estimated the regional disparities in the progress of health-related SDG indicators by grouping four high-HDI countries and six low-HDI countries and plotted the attainment index in 2000, 2015 and 2030.

### 2.6. Data quality

To ensure the quality of data, we compared the GBD Study 2,021 data with other official sources of data in ASEAN, which reported similar indicators, including but not limited to national surveillance, survey, disease registry, and death registry. The cross-validated method improved the quality and reliability of our data. To enhance the methodological robustness of the inclusion, categorization, measurement, and attainment of each indicator, we organized meetings with technical experts from WHO to reach practical consensus on the use of the data that are most comparable to statistics reported by WHO, Demographic and Health Surveys (DHS) and other nationally representative data sources. More details were described in S1 Appendix. Moreover, from January 2023 to October 2024, we reviewed the literature, reports from World Bank and WHO, policy documents, and national survey reports to verify data on key health indicators. Data analysis was conducted using a structured computational framework developed in Microsoft Excel (Microsoft Corporation, Redmond, WA, USA) and Stata 16 (StataCorp, College Station, TX). The completed GATHER checklist is provided in S2 Checklist GATHER checklist to ensure compliance with transparent reporting standards.

## 3. Results

### 3.1. Overall progress and attainment

**Fig 1:**
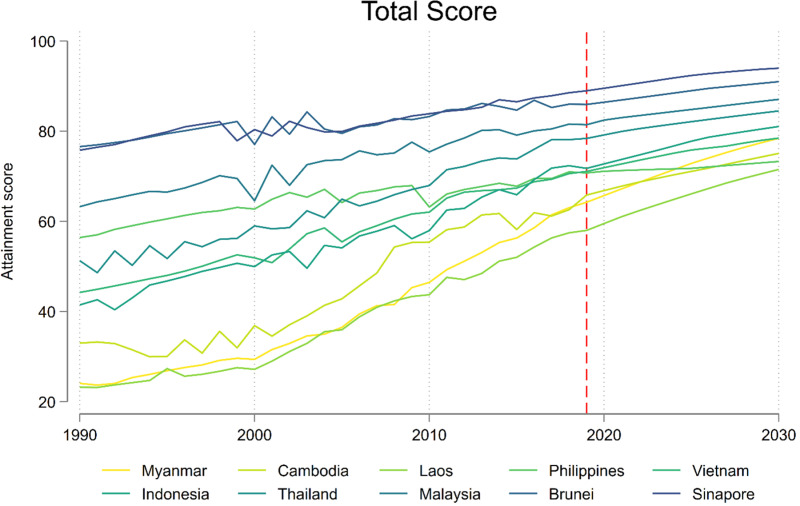
Aggregated trends of SDG indicators from 1990 to 2030 over ASEAN member countries. Note: MCH denotes maternal and child health; NCD denotes non-communicable diseases; UHC denotes universal health coverage. The dash red lines indicate the year 2017, distinguishing between past estimates and future projections. In the legend, the countries are ranked in human development index in 2015, with darker blue indicating higher index.

**Fig 2:**
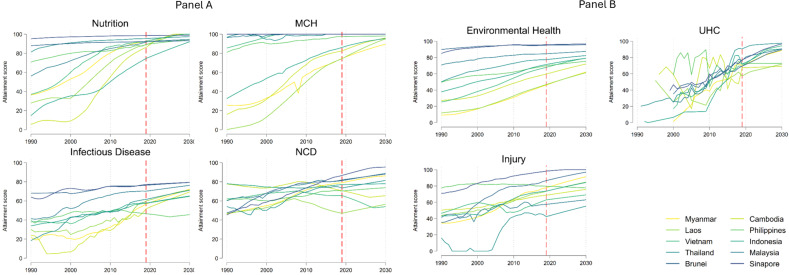
Thematic trends of SDG indicators from 1990 to 2030 over ASEAN member countries. Note: MCH denotes maternal and child health; NCD denotes non-communicable diseases; UHC denotes universal health coverage. The dash red lines indicate the year 2017, distinguishing between past estimates and future projections. In the legend, the countries are ranked in human development index in 2015, with darker blue indicating higher index. The detailed trends for each specific indicator are presented in Fig A1 in S1 Appendix.

**Fig 3:**
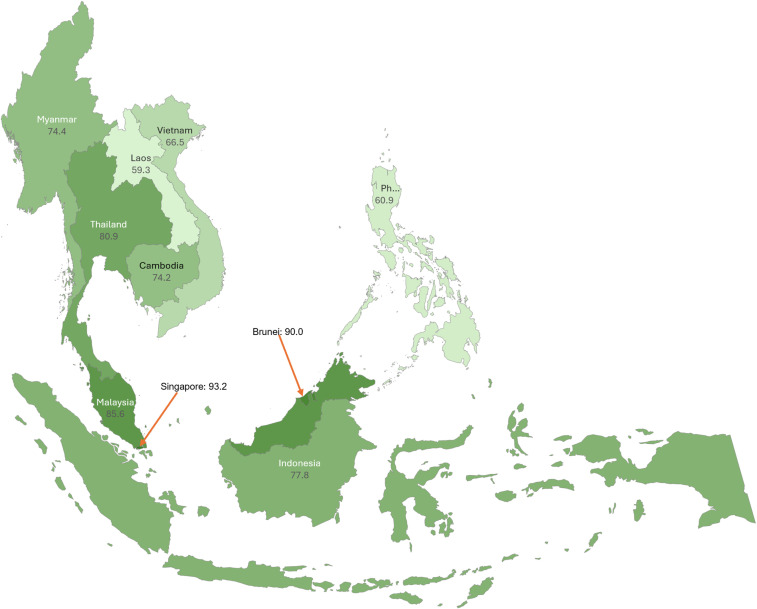
Geographic plot of overall attainment index in ASEAN in 2030. Note: Areas in green indicate a value ranging from 0 to 100. Darker green indicates a higher value. Map data courtesy of https://www.microsoft.com/en-us/maps; Available under a Creative Commons Attribution (CC BY) license.

Between 1990 and 2030, ASEAN member countries are projected to make robust and positive progress towards the SDG targets (Fig 1). The overall positive trends are mainly driven by the progress in nutrition, MCH, infectious disease, and environmental health. However, the projected progress in NCDs, UHC and road injuries varies across member countries (Fig 2A, 2B). Based on these trends, we estimated the total scores for SDG indicators in 2030 (Fig 3). Singapore (93.2 versus 85.0 in 2015) and Brunei (90.0 versus 83.2 in 2015) are estimated to achieve their SDG targets at the aggregate level in 2030. Malaysia (85.6 versus 77.3 in 2015) and Thailand (80.9 versus 70.0 in 2015) are strong performers, followed by the other 6 member countries including Indonesia (77.8 versus 63.8 in 2015), Myanmar (74.4 versus 51.3 in 2015), Cambodia (74.2 versus 48.5 in 2015), Vietnam (66.5 versus 63.5 in 2015), Philippines (60.9 versus 63.0 in 2015), and Laos (59.3 versus 47.6 in 2015). As a block, ASEAN is estimated to achieve a total score of 76.2 in 2030, as compared with 65.3 in 2015.

### 3.2. Topic by thematic areas

**Fig 4:**
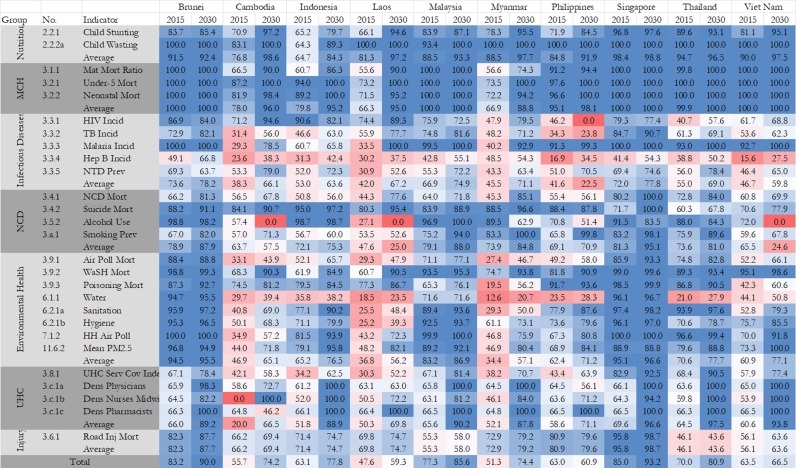
The colorful MAP for attainment index by country/indicators (2015 versus 2030). Note: MCH denotes maternal and child health; NCD denotes non-communicable diseases; UHC denotes universal health coverage. Grids in red indicate a value less than 60 and darker further away from 60. Grids in light blue indicate a value bigger than 60 but smaller than 90. Darker nearer to 90. Grids in dark blues indicate a value bigger than 90.

**Fig 5:**
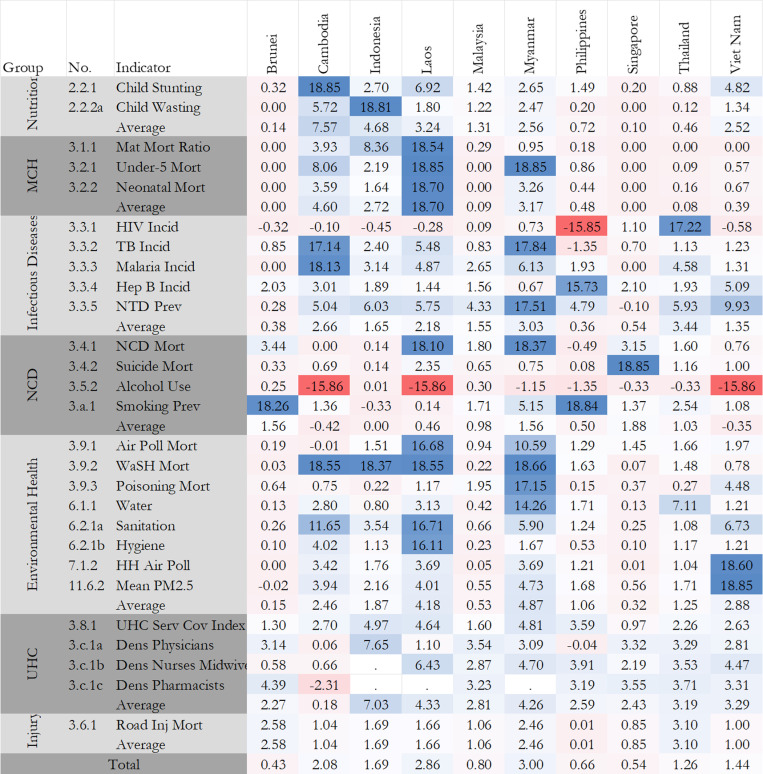
Annual change rate in the attainment index of health-related SDG indicators between 1990 and 2030. Note: Grids in red indicate an increase lower than the 50th percentile of all annual change rates from 1990 to 2030, while grids in blue indicate an increase higher than the 50th percentile of all annual change rates from 1990 to 2030. Grids in while indicate the 50th percentile of all annual change rates from 1990 to 2030 and darker colors indicate further away from the 50th percentile. The dots indicate missing historical values between 1990 and 2030.

We disaggregated the overall attainment score into 7 thematic areas: (i) nutrition, (ii) maternal, child and reproductive health, (iii) infectious diseases, (iv) non-communicable diseases, (v) environmental health, (vi) universal health coverage, and (vii) road injuries. For nutrition and MCH, 9 member countries (Nutrition: Brunei, Cambodia, Laos, Malaysia, Myanmar, Philippines, Singapore, Thailand, Vietnam; MCH: Brunei, Cambodia, Indonesia, Laos, Malaysia, Philippines, Singapore, Thailand, Vietnam) are projected to achieve their targets by 2030; 7 member countries (Brunei, Indonesia, Malaysia, Myanmar, Singapore, Thailand, Vietnam) are projected to achieve or close to their target for UHC in 2030. Conversely, no countries are project to achieve their targets for infectious diseases in 2030; only Singapore is projected to achieve its target for NCDs; only Brunei and Singapore are projected to achieve their target for environmental health in 2030; and only Singapore is projected to achieve their target for road injuries in 2030. The details can be found in Fig 4. In the following section, we explored detailed composite for each thematic area, and also calculated the annual change rate to understand the speed of progress (Fig 5).

#### 3.2.1. Nutrition.

Estimates of progress in nutrition include the prevalence of stunting and prevalence of wasting among children under 5. As the GBD 2,021 dataset does not release the obesity indicator it is not included in this assessment.

We find overall positive progress in nutrition in ASEAN (2015: 86.4, 2030: 97.1). Among children aged 5 years and younger, the stunting rate reduced from 5.5% to 3.2% and wasting reduced from 3.3% to 2.0% in Singapore from 1990 to 2015. Similarly, the stunting rate reduced from 29.7% to 18.7% and wasting reduced from 7.9% to 4.6% in Myanmar from 1990 to 2015. We projected an increase in the attainment score for child undernutrition for all member countries in the coming decade if current trends continue. Cambodia, Laos, and Vietnam are three countries ranking top 3 in the progress. In this case, the best performer is projected to be Singapore (98.8) compared with the worst case of Indonesia (84.3) in 2030. The details for other countries can be found in Fig 4.

#### 3.2.2. Maternal, child and reproductive health.

Indicators for MCH include the maternal mortality ratio in women aged 10−54 years, under-5 mortality rate, and neonatal mortality rate.

ASEAN overall is projected to continually make good progress in MCH (2015: 88.9, 2030: 97.4). For example, maternal mortality ratio, under-5 mortality rate, and neonatal mortality rates in Singapore have decreased to very low levels, reaching 4.2/100,000, 2.0‰, and 0.96‰ in 2015, and the rates are projected to be 2.1/100,000, 1.2‰, and 0.67‰ in 2030; similarly in Myanmar the rates reach 183.8/100,000, 51.6‰, and 22.7‰ in 2015, and are projected to be 128.3/100,000, 24.6‰, and 14.2‰ in 2030. Laos, Cambodia, and Myanmar are three countries ranking top 3 in the progress. In this case, the best performer is projected to be Brunei, Malaysia, Singapore, Thailand, and Vietnam (100.0) compared with the worst case of Myanmar (88.8) in 2030. The challenge in Myanmar is driven by the comparable slow progress in maternal mortality ratio (74.3 in 2030). The details for other countries can be found in Fig 4.

#### 3.2.3. Infectious diseases.

Indicators for infectious diseases include age-standardized rate of new HIV infections, tuberculosis cases, malaria cases, hepatitis B incidence, and age-standardized prevalence of the sum of 15 neglected tropical diseases.

The attainment of infectious diseases in ASEAN is projected to be mixed (2015: 56.9, 2030: 69.4). New HIV/AIDS cases increased steadily in a majority of ASEAN member countries, while cases of TB, malaria, hepatitis B, and neglected tropical diseases decreased in the past decade. For example, in Myanmar in 2015, the incidence of HIV/AIDS was 24.5/100,000, followed by TB at 202.0/100,000, malaria at 821.1/100,000, and hepatitis B at 507.1/100,000. The incidence of the sum of 15 NTDs (i.e., soil-transmitted helminths, leprosy, lymphatic filariasis and trachoma) was estimated to be 17,117.8/100,000. By 2030 in Myanmar, rates are projected to be: 8.6/100,000 for HIV/AIDS, 110.6/100,000 for TB, 115.7/100,000 for malaria, 410.2/100,000 for hepatitis B, and 28,475.6/100,000 for NTDs. However, in Singapore, the incidence of HIV/AIDS was 8.7/100,000 in 2015 but is projected to be 9.6/100,000 in 2030, although Singapore is projected to perform well in the control of other infectious diseases.

Progress towards infectious disease targets were greatest in Thailand, Myanmar, and Cambodia. In this case, the best performer is projected to be Brunei (78.2) compared with the worst case of Philippines (22.5) in 2030. The details for other countries can be found in Fig 4. The low attainment score for Philippines is mainly driven by the poor control of HIV, TB and hepatitis b in the country. In addition, there are great challenges of ending HIV/AIDs, TB, hepatitis B and NTDs in the region, although major member countries are estimated to achieve their targets for malaria.

#### 3.2.4. Non-communicable diseases.

Indicators for non-communicable diseases include age-standardized death rate due to cardiovascular disease, cancer, diabetes, and chronic respiratory disease in populations aged 30–70, age-standardized death rate due to self-harm, risk-weighted prevalence of alcohol consumption, and age-standardized prevalence of current smoking in populations aged 10 and older.

The attainment of NCDs in ASEAN is also projected to be mixed (2015: 73.0, 2030: 76.7). For example, in Myanmar, the age-standardized mortality of the four major NCDs was 6176.8/100,000 in 2015 and is projected to decrease to 4596.1/100,000 in 2030; the suicide mortality rate has decreased over the past decade, reaching 3.9/100,000 in 2015 and is projected to reach 2.9/100,000 in 2030; the smoking prevalence was11.7% in 2015 and is projected to be 8.4% in 2030. However, the prevalence of alcohol use is projected to increase from 6.4% in 2015 to 10.9% in 2030. Correspondingly, in Singapore, the age-standardized mortality of the four major NCDs was 1733.2/100,000 in 2015 and is projected to decrease to 973.3/100,000 in 2030; the suicide mortality rate was 7.8/100,000 in 2015 and is projected to reach 4.8/100,000 in 2030; the smoking prevalence was 11.2% in 2015 and is projected to be 8.8% in 2030. However, the prevalence of alcohol use is projected to increase from 8.8% in 2015 to 9.9% in 2030.

Singapore, Brunei, and Myanmar are three countries ranking top 3 in the progress of NCDs. In this case, the best performer is projected to be Singapore (95.1) compared with the worst case of Vietnam (24.6), Laos (25.0), and Philippines (28.3) in 2030. The details for other countries can be found in Fig 4. We note that the key driver for the low attainment index in NCDs for Cambodia, Laos and Vietnam is the growing prevalence of alcohol consumption, although Laos also faces the challenge of a rise in smoking prevalence. ASEAN faces major challenges in reducing the mortality related to NCDs and controlling its risk factors.

#### 3.2.5. Environmental health.

Indicators for environmental health included age-standardized death rate attributable to household air pollution and ambient air pollution, attributable to unsafe water, sanitation, and hygiene, due to unintentional poisonings, and 4 indicators environmental risk factors such as risk-weighted prevalence of populations using unsafe or unimproved water sources, of populations using unsafe or unimproved sanitation, of populations without access to a handwashing facility, of household air pollution, and population-weighted mean levels of fine particulate matter smaller than 2.5 microns in diameter.

ASEAN achieved robust, despite sometimes slow, progress in environmental health (2015: 68.4, 2030: 79.1). For example, in Myanmar, the rate of age-standardized death rate attributable to indoor air pollution and ambient air pollution, age-standardized death rate attributable to unsafe water, sanitation, and hygiene, and age-standardized death rate due to unintentional poisonings was 271.3/100,000, 20.0/100,000, and 1.4/100,000 in 2015 and is projected to be 188.7/100,000, 7.8/100,000, and 0.87/100,000 in 2030. In addition, the risk-weighted prevalence of populations using unsafe or unimproved water sources, using unsafe or unimproved sanitation, and without access to a handwashing facility, the risk-weighted prevalence of household air pollution, and the population-weighted mean levels of fine particulate matter smaller than 2.5 microns in diameter (PM2.5) were 62.8%, 62.8%, 20.7%, 42.1%, and 59.8% in 2015 and are projected to be 57.1%, 44.5%, 14.4%, 19.1%, and 41.0% in 2030. Singapore has similar trends but with better performance.

Myanmar, Laos, and Vietnam are three countries ranking top 3 in the progress of environmental health. In this case, the best performer is projected to be Singapore (96.6) compared with the worst case of Laos (56.2) in 2030. The details for other countries can be found in Fig 4. ASEAN also faces significant challenges in reducing the environment related to mortality and controlling its risk factors.

#### 3.2.6. Universal health coverage.

We have 4 indicators for universal health coverage, including coverage of essential health services, density of medical doctors, of nursing and midwifery personnel, and of pharmacists.

ASEAN’s progress in UHC is projected to improve toward 2030 (2015: 58.7, 2030: 86.2). For example, in Myanmar, the attainment index in UHC index is projected to significantly increase from 53.0 in 2015 to 77.8 in 2030; the number of medical doctors, nursing and midwifery personnel, and pharmacists is projected to increase from 6.2/10,000, 10.4/10,000, and 0.34/10,000 in 2015 to 11.2/10,000, 14.0/10,000, and 9.4/10,000, respectively in 2030. Similarly, in Singapore, the attainment index in UHC index is projected to significantly increase from 87.0 in 2015 to 94.3 in 2030; the number of medical doctors, nursing and midwifery personnel, and pharmacists is projected to increase from 22.1/10,000, 59.5/10,000, and 4.7/10,000 in 2015 to 33.8/10,000, 84.3/10,000, and 8.58/10,000 in 2030.

Indonesia, Laos, and Myanmar are three countries ranking top 3 in the progress of UHC. In this case, the best performer is projected to be Thailand (97.5) compared with the worst case of Cambodia (66.5) in 2030. We estimate 4 of 10 member countries can achieve their goals for UHC, 3 out of 10 member countries are near the threshold, while 3 out of 10 member countries follow closely. The details for other countries can be found in Fig 4.

#### 3.2.7. Road injuries.

ASEAN’s progress in road injury mortality is projected to improve based on its historical progress (2015: 69.7, 2030: 79.9). For example, in Myanmar, the age-standardized death rate due to road injuries was 16.2/100,000 in 2015 and is projected to be 10.6/100,000 in 2030; as a comparison in Myanmar, the age-standardized death rate due to road injuries was 2.7/100,000 in 2015 and is projected to be 0.87/100,000 in 2030. Thailand, Brunei, and Myanmar are three countries ranking top 3 in the progress of road injury. In this case, the best performer is projected to be Singapore (98.7) compared with the worst case of Malaysia (58.0) in 2030. The details for other countries can be found in Fig 4.

### 3.3. Regional disparities

**Fig 6:**
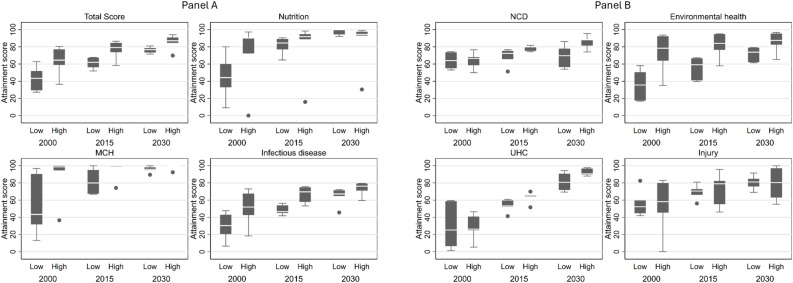
Disparities in attainment index over years and countries. Note: MCH denotes maternal and child health; NCD denotes non-communicable diseases; UHC denotes universal health coverage. HDI denotes human development index. Low HDI countries include Philippines, Indonesia, Vietnam, Laos, Cambodia, Myanmar using 0.800 as a cutoff. High HDI countries include Singapore, Brunei, Malaysia, Thailand. The center line inside the box represents the median. The box itself represents the interquartile range (IQR). Whiskers extend to the smallest and largest values within 1.5 × IQR from Q1 (25th percentile) and Q3 (75th percentile). Dots placed past the line edges to indicate outliers.

The disparities in the 27 selected health-related SDG indicators among ASEAN member countries are estimated to be narrowed from 2000 to 2030 overall (Fig 6A, 6B), despite the pattern changing across thematic areas. Disparities in nutrition, MCH, infectious diseases, and road injury were narrowed from 2000 to 2015, with low-HDI countries estimated to catch up high-HDI countries by 2030.

For example (Fig A2 in S1 Appendix), no apparent disparities in the prevalence of child stunning and child wasting are observed in 2030; all late comers are projected to achieve a comparable level to advanced economies in maternal mortality, under-5 mortality and neonatal mortality in 2030; and low-HDI countries are projected to perform better than high-HDI countries in the control of HIV and road injury in 2030. However, regional disparities in NCDs, environmental health, and UHC are largely projected to persist or worsen. For example, slower progress in reducing alcohol consumption was evident among low-HDI countries while being stable among high-HDI countries, resulting in growing disparities in the indicator. Low-HDI countries are also projected to have slower progress than high-HDI countries in the control of smoking, reducing air pollution mortality, reducing unsafe water use, and improving physician-to-population density.

## 4. Discussion

In the assessment of ASEAN’s progress towards the 2030 health-related SDGs, our results indicated robust and positive improvements in the aggregated SDG scores in every member country till 2030. Two countries, Singapore and Brunei, are projected to achieve their overall targets. Nine out of 10 member countries are projected to achieve their targets for nutrition and MCH, seven of 10 are projected to achieve or very close to their targets for UHC, whereas two of 10 are projected to achieve their targets for environmental health and road injuries, and just one of 10 for NCDs. No country is projected to achieve infectious disease-related targets by 2030.

The significant progress in nutrition, MCH, and UHC across ASEAN countries is very encouraging and can be the result of strong political commitment, comprehensive health strategies, and international support. Particularly, ASEAN countries have shown strong political commitment towards UHC, ensuring that healthcare is accessible to all citizens, regardless of their socio-economic status [49]. Member countries have adopted national nutrition strategies, including micronutrient supplementation, breastfeeding promotion, and food fortification, that focus on maternal, infant, and young child nutrition [50]. In addition, international initiatives like the Scaling Up Nutrition have played a crucial role in mobilizing global efforts towards maternal and child health, emphasizing the first 1,000 days from conception to the child’s second birthday as critical for nutrition interventions [51]. Moreover, successful efforts have incorporated sectors beyond health, such as agriculture, education, and social protection, to address the broader social determinants of health. Investments in women’s education, sanitation, and food security have contributed significantly to reducing malnutrition and child mortality [52]. Last but not least, ASEAN’s regional cooperation in health policy has also enhanced resource sharing and capacity-building across member countries, including efforts to align national policies with global goals and increase access to essential health services, leading to improved maternal and child health outcomes [53].

The slow to moderate progress in NCDs, infectious diseases, environmental health, and road injuries reflects both achievements and ongoing challenges. ASEAN is facing the double burden of NCDs and infectious diseases like many other developing countries. ASEAN is experiencing a rapid transition from active lifestyles and traditional healthy fresh food diets to ever-increasing consumption of highly processed food and drinks heavy in unhealthy fat, sugar and salt [54]. Rapidly increasing overweight among children is an alarming result in the region [55]. Combined with the high prevalence of undernutrition, the malnutrition, whether under or over, is also happening in middle-income countries such as Indonesia, Malaysia, the Philippines and Thailand [56]. Both undernutrition and overweight contribute to the increased burden of NCDs and cardiovascular diseases. From 1990 to 2017, Vietnam, Cambodia and Laos witnessed a 40%–90% increase in harmful alcohol consumption, some of the highest in Asia and the world [57]. As one of the most densely populated areas worldwide, ASEAN indeed faces the challenge of population aging.

Moreover, ASEAN is challenged to respond to pollution and environmental health problems. The geographical location near to the Pacific Ocean, high migration rates, deforestation, and increased food production make ASEAN member countries major hotspots vulnerable to outbreaks of emerging infectious diseases [58]. The region has already borne the highest overall burden of hepatitis B, with 59% of those living with chronic hepatitis B, 26% of new infections, and 79% of deaths. Indonesia has the second highest TB burden worldwide and a fast-growing HIV epidemic [59]. In addition, the region has a great liability for the world’s road traffic injuries, mainly due to the increasing number of motor vehicles and the poor development of cities [60]. These transitions are further complicated by the region’s vulnerability to natural hazards. Southeast Asia is particularly vulnerable to the effects of climate change and ASEAN’s local sustainable development challenges can be magnified by the impacts of climate change at a global level [61].

ASEAN countries exhibit significant diversity in their health systems, economic development, and societal structures. For example, Thailand, Indonesia, Vietnam and Philippines have developed social health insurance systems to ensure the access to essential healthcare [62], while Malaysia and Brunei primarily rely on universal access to good-quality public-sector services [63]. There is also difference in supporting the poor and vulnerable populations or the all [64–66], therefore access to healthcare services varies greatly [67]. Singapore and Thailand have made substantial strides toward UHC, while Cambodia, Laos, and Myanmar lag due to lower government spending and fragmented health system [49]. Inequities in financial protection persist, making it difficult to achieve full UHC although member countries have implemented health financing reforms. Indonesia and Philippines struggle with ensuring affordable access for poorer populations [68] because of low levels of health expenditure and reliance on out-of-pocket payments. Member countries face supply-side constraints, such as insufficient healthcare workers and inadequate infrastructure, particularly in rural and remote areas [69]. The inequitable access to affordable and quality healthcare services between and within ASEAN member countries can lead to disparities in UHC, MCH, infectious diseases, NCDs and beyond.

Our results are very encouraging that in ASEAN, the disparities in SDG indicators are estimated to be narrowed from 2000 to 2030 overall. This reduction of regional disparities was mainly driven by the catch-up of low-HDI countries in nutrition, MCH, infectious diseases, and road injuries. Addressing the remaining disparities in NCDs, environmental health, and UHC will be more challenging. ASEAN’s regional integration offers great opportunities for health sector improvements, but it also risks widening health inequalities, as wealthier populations may benefit more from liberalized health trade policies, and migration of healthcare workers can exacerbate shortages in public sector [49]. In this sense, ASEAN shares very similar challenges with its close neighbors, China and India, in achieving the health-related SDGs [44,70]. ASEAN is characterized by much diversity in terms of demographics, geography, social culture, economic development, political systems and health profiles [49]. This diversity offers opportunities for collaboration and knowledge sharing, enabling faster progress towards SDGs, particularly helping low-HDI countries to catch up. However, it can become more difficult to further progress in SDGs when the latecomers reach a reasonably high level.

This evidence-based study systematically analyzes ASEAN’s progress toward the health-related SDGs and highlights the specific challenges. We used estimates from the GBD Study 2021 and publicly available data to perform quantitative analysis and projections. Extensive literature and policy reviews were conducted to draw a comprehensive picture of AESAN’s progress in health-related SDGs. The presentation of an attainment index ranging from 0 to 100 is novel by communicating a direct message to policymakers and a broader audience. In this sense, our methodology innovation has the potential to pin to a monitoring system that tracks a country’s attainment of health-related SDGs. Furthermore, the results of this study can serve as strong evidence for the local governments and the international community to (i) understand the progress, challenges and gaps toward achieving the health-related SDGs in ASEAN, and (ii) help with effective health policymaking.

This study acknowledges several limitations. First, the current release of GBD 2021 does not include many SDG indicators, and additional reliable data is scarce to present a more comprehensive picture with additional SDG indicators (i.e., obesity, prevalence of sexual violence, death rate due to interpersonal violence), and the associated gender disparities. However, future studies could consider including more indicators in the analysis, especially when data are available for cross validation. Second, national data for some SDG indicators are defined differently from the official ones by the UN (i.e., the rate of child stunting and wasting). These inconsistencies may make international comparisons challenging. Third, we carefully calibrated projection models through comprehensive comparisons and using historical trajectories to predict future changes. Radical political commitments and disruptive technological interventions that are not comparable to historical events cannot be captured by our model. Also, our methodology does not account for ceiling effects. For example, it can be more difficult for a country to further reduce its maternal mortality rates when it reaches a low level. Fourth, the GBD 2021 forecasting model are more comprehensive using past trends together with the composite covariate, socio-demographic index, which captures the socioeconomic and demographic development of a country, to inform future trend forecasts. We did not include these variables in the projection model again to introduce extra uncertainties.

In conclusion, paying attention to ASEAN’s progress in health-related SDGs is crucial to understand global economic trends, regional integration efforts, and the diverse opportunities in Southeast Asia. The region’s trajectory has implications for trade, investment and sustainable development at both regional and global levels. Our results show that ASEAN has made good progress in improving population health, especially for the health of children and women and UHC. For areas where ASEAN has already achieved the SDG targets, the governments should synthesize the experiences and best practices that can be applied to member countries that need to catch up. These experiences and best practices could also be disseminated to other low-and-middle-income countries that are developing their national strategies in advancing the health-related SDGs.

However, ASEAN faces significant challenges to achieve their health-related SDG targets by 2030 in infectious disease, NCDs, environmental health, and road injuries. Moreover, epidemiological transitions and worsening environmental threats would pose extra challenges for ASEAN to achieve their sustainable development goals. Effective responses to the double burden of infectious diseases and NCDS, pollution, environmental risks, and road injuries are important. ASEAN needs to take concerted actions to overcome challenges and achieve the health-related SDGs. To make good progress as a whole, ASEAN should also take a series of concerted actions to invest more in public and merit goods and services for health and to address regional disparities. Leveraging holistic strategies to reduce disparities in health-related SDGs will introduce significant gains for every country and beyond.

## Supporting information

S1 Appendix Detailed methods and supplementary exhibitions. (DOCX)

S2 Checklist GATHER checklist. (DOCX)

## Abbreviations

**:**
ASEAN: association of Southeast Asian nations
DHS: demographic and health surveys
GBD: global burden of disease
GDPpc: per capita gross domestic product
HDI: human development index
MDG: millennium development goals
MCH: maternal, child and reproductive health
NCDs: non-communicable diseases
SDGs: sustainable development goals
WHO: world health organization
