# Supplementary material for: Progressing towards the 2030 health-related SDGs in ASEAN: A systematic analysis
Source: PLoS Med. 2025 Apr 21;22(4):e1004551. doi: 10.1371/journal.pmed.1004551 (PMC12058153; doi:10.1371/journal.pmed.1004551)
Supplement: S1 Appendix — (DOCX) [file pmed.1004551.s001.docx]

**Progressing towards the 2030 health-related SDGs in ASEAN: A systematic analysis**

Contents

[Appendix 1 Detailed methods used for the study 2](#_Toc194234170)

[Figure A1 The trend of specific SDG indicators 1990-2030 7](#_Toc194234171)

[1.1 Specific indicator in nutrition 7](#_Toc194234172)

[1.2 Specific indicator for MCH 8](#_Toc194234173)

[1.3 Specific indicator for infectious disease 9](#_Toc194234174)

[1.4 Specific indicator for NCD 11](#_Toc194234175)

[1.5 Specific indicator for environmental health 12](#_Toc194234176)

[1.6 Specific indicator for UHC 14](#_Toc194234177)

[1.7 Specific indicator in injury 15](#_Toc194234178)

[Figure A2 Regional disparities for specific SDG indicators 16](#_Toc194234179)

[2.1 Specific indicators for nutrition 16](#_Toc194234180)

[2.2 Specific indicators for MCH 17](#_Toc194234181)

[2.3 Specific indicators for infectious 18](#_Toc194234182)

[2.4 Specific indicators for NCD 20](#_Toc194234183)

[2.5 Specific indicators for environmental health 21](#_Toc194234184)

[2.6 Specific indicators for UHC 23](#_Toc194234185)

[2.7 Specific indicators for injury 24](#_Toc194234186)

[Table A1 GBD 2021 estimates and uncertainty intervals in baseline (2015) 25](#_Toc194234187)

# Appendix 1 Detailed methods used for the study

1. **Justifications for the exclusion of nine indicators**

We have selected 27 key health-related SDG indicators based on following criteria: a) direct relevance to health improvement in ASEAN context; b) clear target value defined by the SDGs or international guidelines/documents such as WHO’s; c) availability of reliable data in ASEAN; and d) potential impacts on health policy development in the near future. In general, the selected indicators are traditionally the main health indicators most commonly used for the monitoring and evaluation of health development progress. We excluded nine indicators as listed below

1.5.1 Disaster mortality

3.7.2 Adolescent birth rate

5.2.1 Intimate partner violence

8.8.1 Disease burden attributable to occupational risks

16.1.1 Homicide

16.1.2 Conflict and terrorism mortality

16.1.3 Violence prevalence

16.2.3 Childhood sexual abuse

17.19.2c Well-certified death registration

While we fully agree that these nine indicators are critically important to the health improvement, we have several concerns or reasons not to include them in this study. For some indicators (1.5.1, 3.7.2, 5.2.1, 8.8.1, 16.1.1, 16.1.2, 16.1.3, and 17.19.2c), we examined and found poor availability of reliable domestic data sources for either GBD 2021 modelling or our comparison for data quality. We exclude the indicators, such as 16.1.1, and 16.2.3, due largely to major political obstacles to their measurement in member countries. Some indicators, e.g. 16.1.1, are not directly related to health. Furthermore, as our attainment index required a clear and well-defined target values, some indicators, such as 3.7.2, do not have any target value in neither the SDGs nor international guidelines.

1. **Sources and use of secondary quantitative data**

This study used many quantitative data from a variety of sources domestically and internationally. Data collected from official sources in ASEAN member countries, including the published survey reports and health statistics yearbooks, were mainly used to describe the current status of the key health indicators. Estimates and projected values of health-related SDGs obtained from the GBD 2021 study were primarily used to calculate the attainment index of health-related SDGs.

1. **Attainment index of health-related SDGs**

To explicitly show the performance of attainment in each indicator, we transformed the absolute value (e.g. death rate) into the Attainment Index of Health-related SDGs. It was an index from 0 to 100 that the higher index marked a better performance. By this means the attainment of each SDG indicator can be measured and compared with that of other indicators. Here is an example of how absolute values were transformed.

Step 1: Setting the 0 value

For the estimate of each indicator from the GBD 2021 study data, we examine the estimates of 1990, 1995, 2000, 2005, 2010, 2015 in 10 ASEAN member countries. These data points were later sorted from the smallest to the largest if a larger value is worse (e.g. death rate), vice versa. To eliminate the influence of the extreme outlier on the allocation of scales, we teased out values larger (smaller) than 1.5 times of the interquartile range (IQR) defined by the range between the 25^th^ percentile and 75^th^ percentile. If the worst value is larger (smaller) than 1.5 times of IQR, then the “0” in the index will be set as the 1.5 times of IRQ; otherwise, the “0” in the index will be the worst value itself.

Step 2: Setting the 100 value

As this index is aiming to measure the attainment of SDG targets, the “100” in the index was defined as the absolute or relative values of the target according to the expression of SDG. The setting of “100” in the index was conducted in four scenarios.

- 1. the SDG has defined an absolute value in the target (e.g. reduce the global maternal mortality ratio to less than 70 per 100,000 live births.). This figure will be set as “100” in the index.
  2. the SDG has defined a relative value in the target (e.g. 1/3 premature NCDs deaths reduction). We used the value in 2015 as the baseline for most of the indicators unless indicated. The target of each province is relative value compared to the baseline of this province. It is to be noted that the absolute values behind the “100” of each province are therefore different.
  3. the SDG has not defined explicit target value, but the WHO has a working target. We adopted the WHO target instead to be the “100” in the index.
  4. the SDG has not defined explicit target value but used a certain expression to describe the extent. If the descriptions contain terms such as “substantially reduce”, we set an “80% relative decrease” as the target, which is the “100” in the index.

Step 3: allocating scales

Considering the wide range and rapid speed of change of indicators about incidence and mortality rates, the scale of these indicators was performed in log space, which is helpful in differentiating and measuring the progress under such conditions.

First, the values of “0” and “100” of index, and values in all estimated and projected years were transformed into their natural log by the below formula.

$$L_{V}=ln(V+e^{4})$$

Where V denotes the absolute value of each indicator and L demotes their modified natural log. To avoid the steep drop of log when the base closes to 0, we add e^4^ on V before calculating the natural log.

As the worst value of each indicator was set as “0” in the index, and the target value was “100”, their modified natural log was used in the scaling in log space.

$$I_{V}=\frac{L_{V}-L_{W}}{L_{T}-L_{W}}*100*k$$

Where $I_{V}$ is the index value; $L_{W}$ is the modified natural log of worst value; $L_{T}$ is the modified natural log of target value; 𝑘 is the reverse coefficient. In some special cases, the worst value of an indicator is better than its target value, 𝑘 will be set as -1 to make sure $I_{V}$ is always positive.

For indicators other than incidence and mortality rate, we calculate them by the below formula without the transformation in log space.

$$I_{V}=\frac{V-W}{T-W}*100*k$$

Where $I_{V}$ is the index value; 𝑉 is the absolute value of indicator; 𝑊 is the worst value of the indicator; 𝑇 is the target value of the indicator; 𝑘 is the reverse coefficient.

To avoid misunderstanding of the index, we also limit the $I_{V}$ in the range from 0 to 100. Any $I_{V}$ larger than 100 or less than 0 will be adjusted to 100 or 0.

Annual change rate

To further compare the progress of each thematic topic in member countries, we calculated the annual change rate of the attainment index from 1990 to 2030 by dividing the increase or decrease of the index by the number of years. To capture and present possible change estimated to occur, we allowed the raw index to go below 0 or beyond 100 and calculated the arithmetic mean of indicators under one thematic topic as its index for annual change rate calculations.

1. **Projection of indicators**

Selection of projection methods

Considering the need to project specific absolute values for indicators, we explored the projection models to find the best-fitted one. We compared five different methods: arithmetic mean change, geometric mean rate of change, alternative geometric mean rate of change, the methods developed by the GBD 2016 SDG collaborators to project health-related SDG indicators attainment in 188 countries, and adjusted GBD method. To select the optimal projection method, we conducted an accuracy test with GBD data of 24 indicators and an evaluation of method generalizability.

We selected the adjusted GBD method because of the following reasons:

- Accuracy: among all candidates, in general this method produced projections with least mean squared error (MSE). Among all indicators, the variance of MSE produced by this method was also the least.
- Generality: This method prevented abnormal projections when being applied on data as percentage, where projections from arithmetic mean change, geometric mean rate of change frequently failed.

Projection of indicators

This adjusted GBD methods following the original methodology to first convert data into logit-space (for percentage data) or natural-log space (for other data) and calculate the annual rate of change. Afterwards, a time-based weight matrix was established to add more weight to rate of change in recent years. The weight of rate of change in a selected year is:

$${Weight}_{year}=\frac{{(year-1990)}^{\omega}}{\sum_{t=1990}^{T} {(t-1990)}^{\omega}}$$

Where T is the last year with available data. The parameter ω was determined with a validity test: we selected the ω that used the first half (1990, 1995, 2000, 2005) of available data to predict the second half (2010, 2015, 2016) most accurately.

Building upon the weight matrix, we calculated the weighted mean annual rate of change and used it to predict the data trend.

The adjustments we made based on the original methodology were: 1) we used weighted mean annual rate of change, instead of median annual rate of change, to predict the data trend after the weight matrix ω was confirmed; 2) the ω was defined by the trend of each indicator in each member country, rather than the global trend. This aimed to improve the validity as the ω from the global trend may not well fit conditions in ASEAN; 3) uncertainty analysis for 95% uncertainty intervals using simulation analysis fall outside of the purview of our study and was not performed. Therefore, we only presented the scaled mean scores without uncertainty intervals, considering its simplicity and the lack of uncertainty analysis results for specific results of indicators related to child nutrition, infectious diseases, NCDs and road injuries.

1. **Qualitative data collection and analysis**

Key informant interviews with relevant stakeholders at both national level and international level were conducted to obtain the knowledge and their opinions on the health achievement and key intervention area in past decades, as well as the challenge in achieving health-related SDGs and domestic targets. We invited a total of around 120 senior government officials/policy-makers, policy advisors, public health professionals, and clinical experts to participate in the interview. Face-to-face/online interviews were conducted, where the interview was recorded under the permission of interviewees.

All recording files were later transcribed into Microsoft Word files. The research team members read through the transcripts and field notes to refine the analysis framework. All data were coded and sorted to summarize the final themes.

# Figure A1 The trend of specific SDG indicators 1990-2030

## 1.1 Specific indicator in nutrition


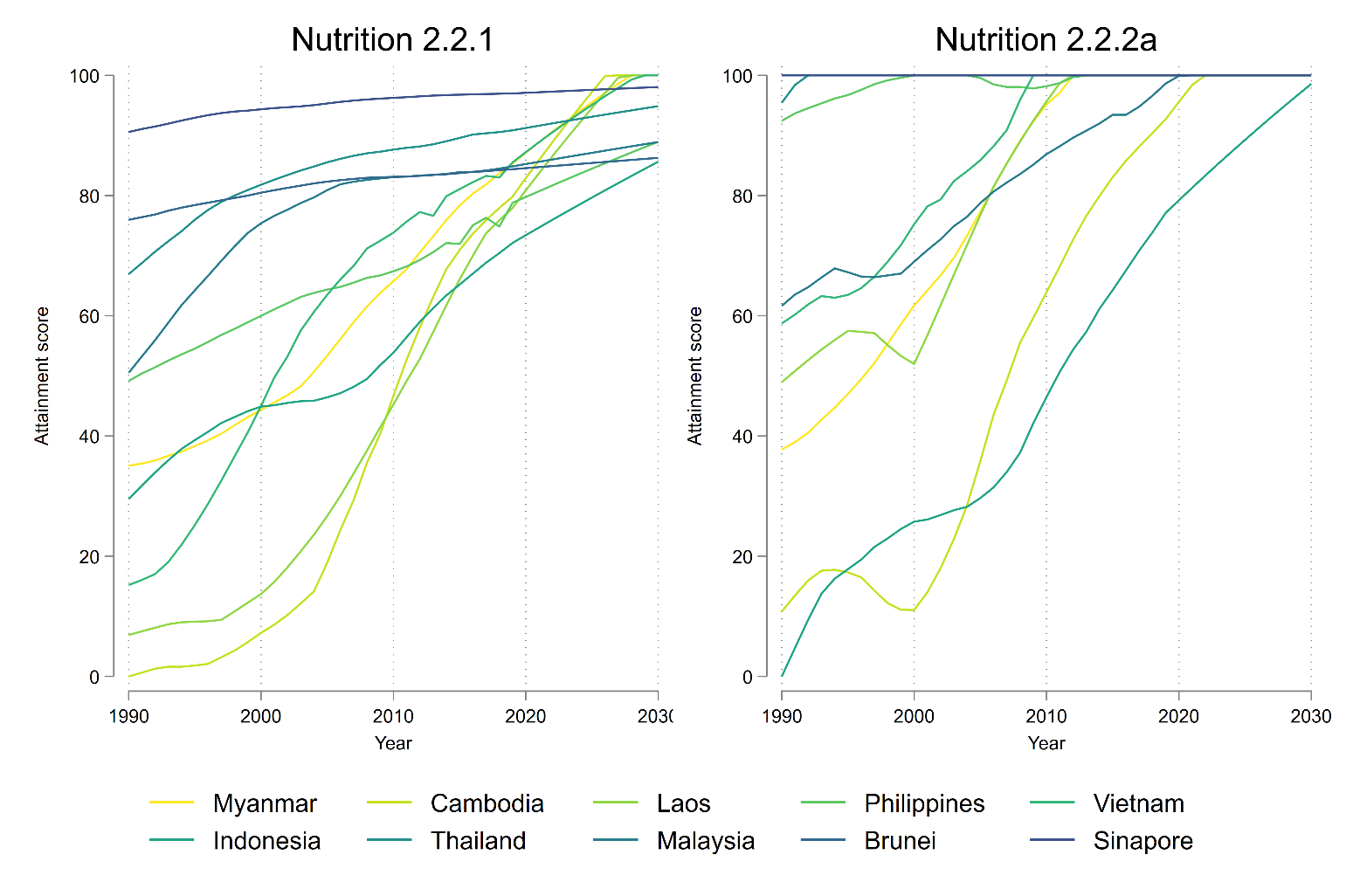


Note: In the legend, the countries are ranked in human development index in 2015, with darker blue indicating higher index.

## 1.2 Specific indicator for MCH


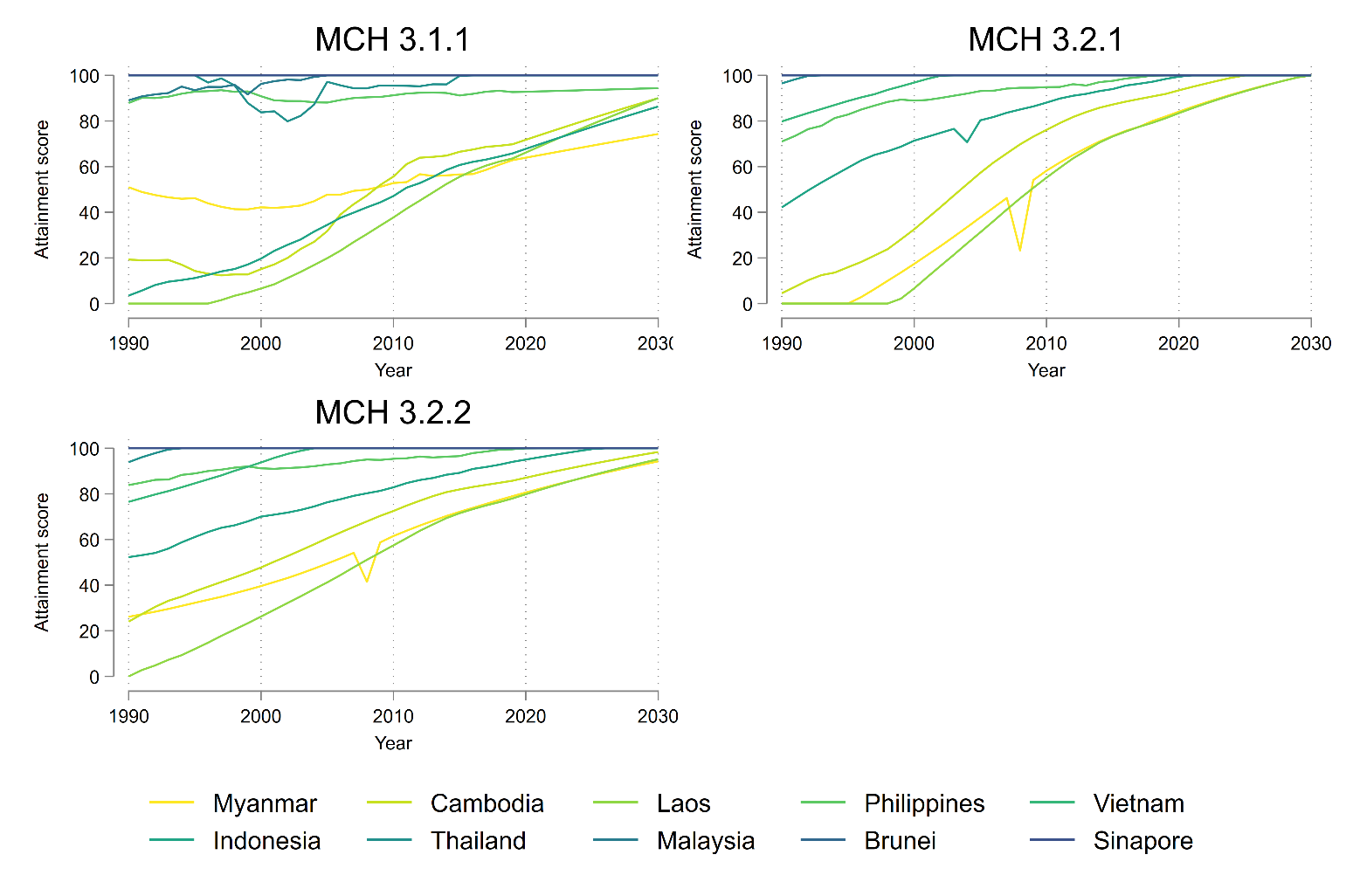


Note: In the legend, the countries are ranked in human development index in 2015, with darker blue indicating higher index.

## 1.3 Specific indicator for infectious disease


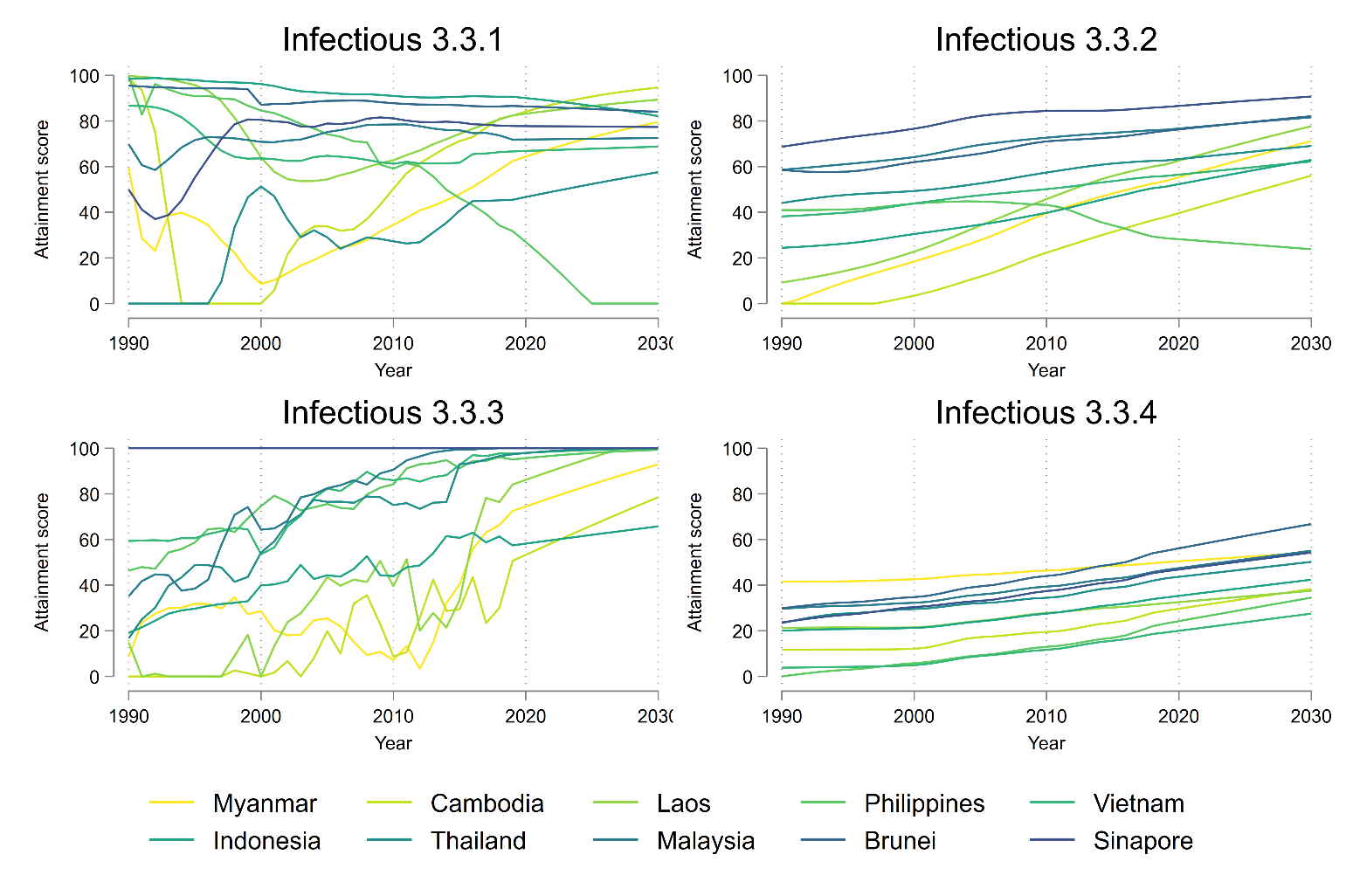


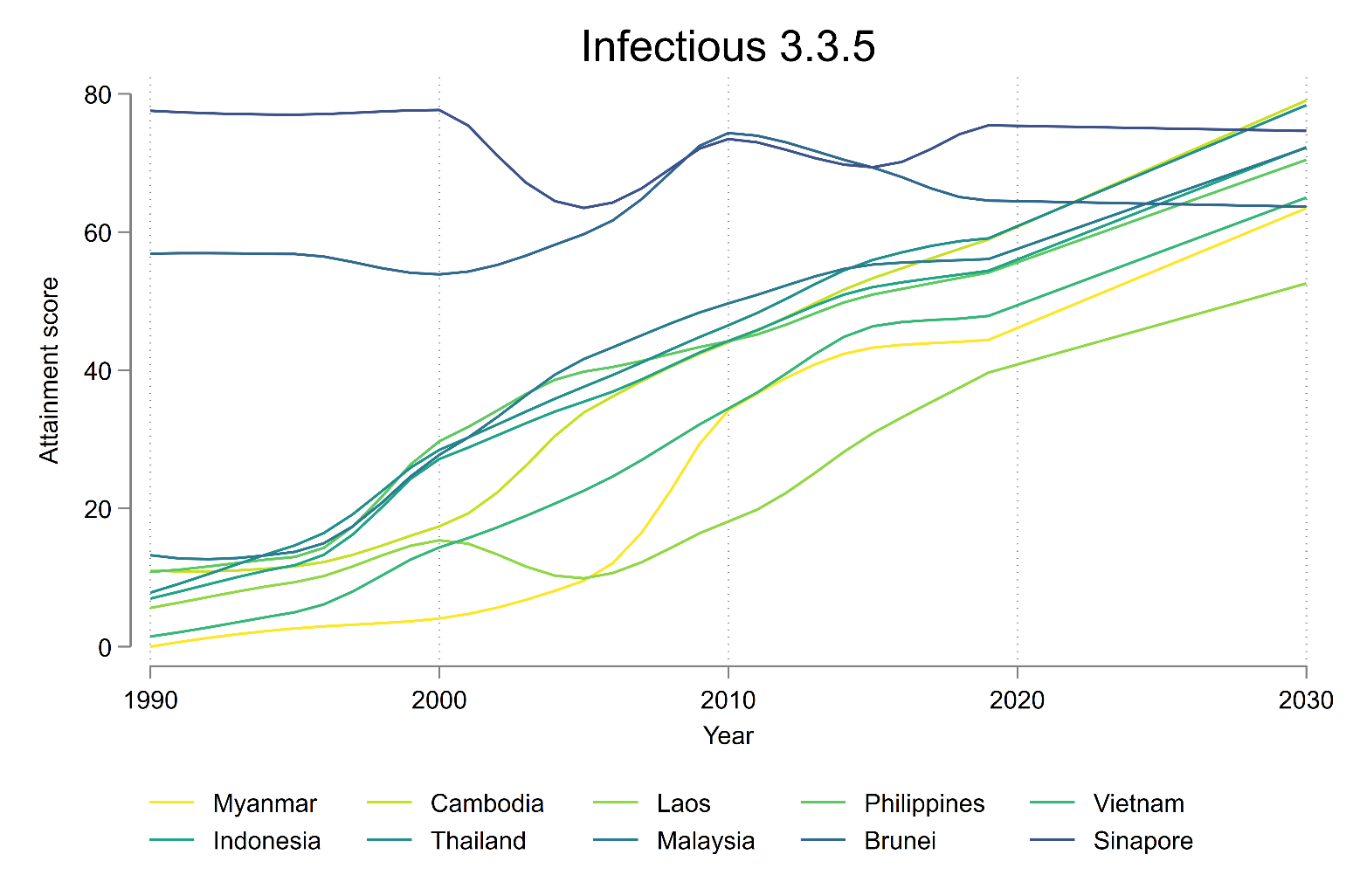


Note: In the legend, the countries are ranked in human development index in 2015, with darker blue indicating higher index.

## 1.4 Specific indicator for NCD


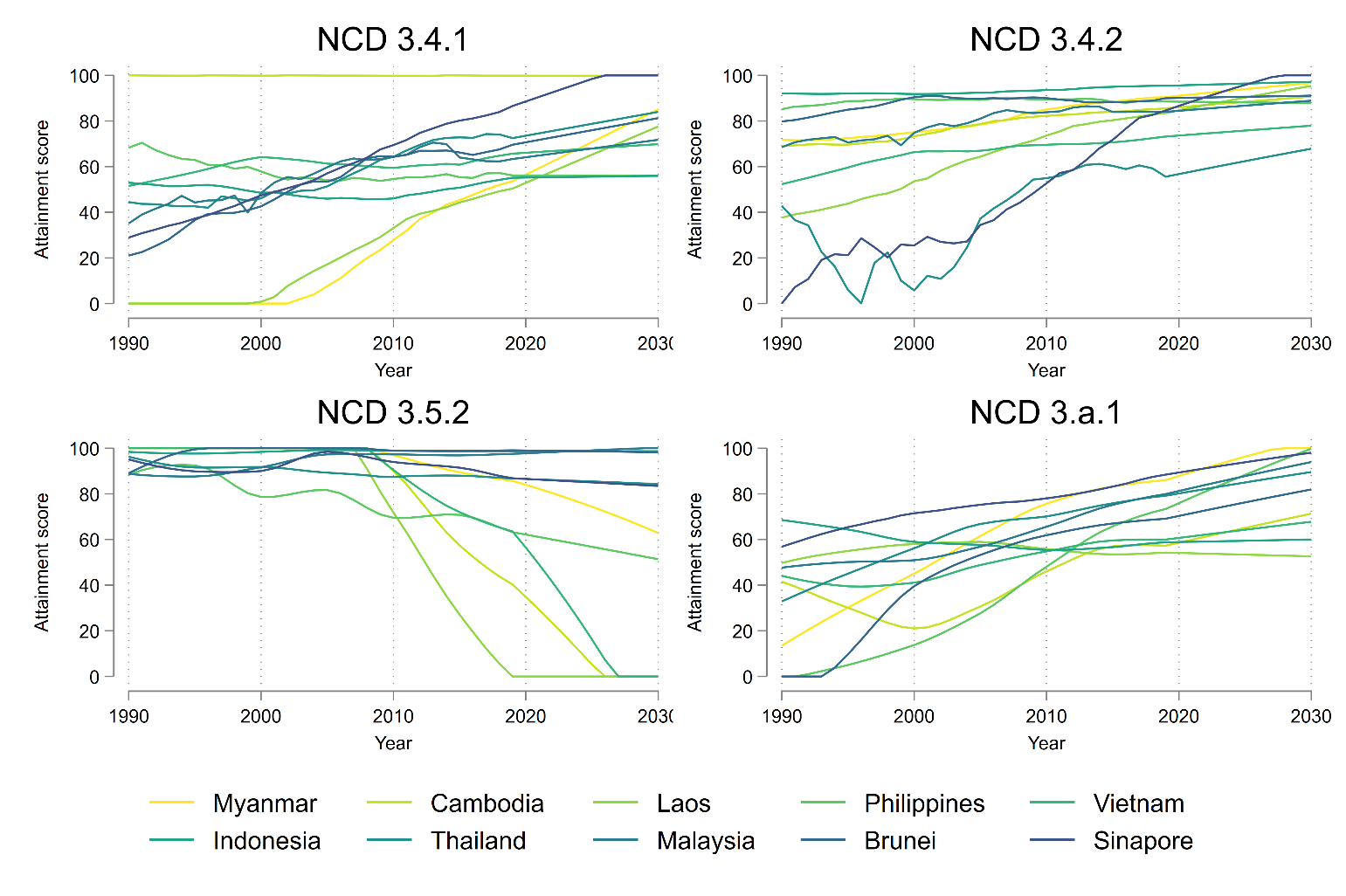


Note: In the legend, the countries are ranked in human development index in 2015, with darker blue indicating higher index.

## 1.5 Specific indicator for environmental health


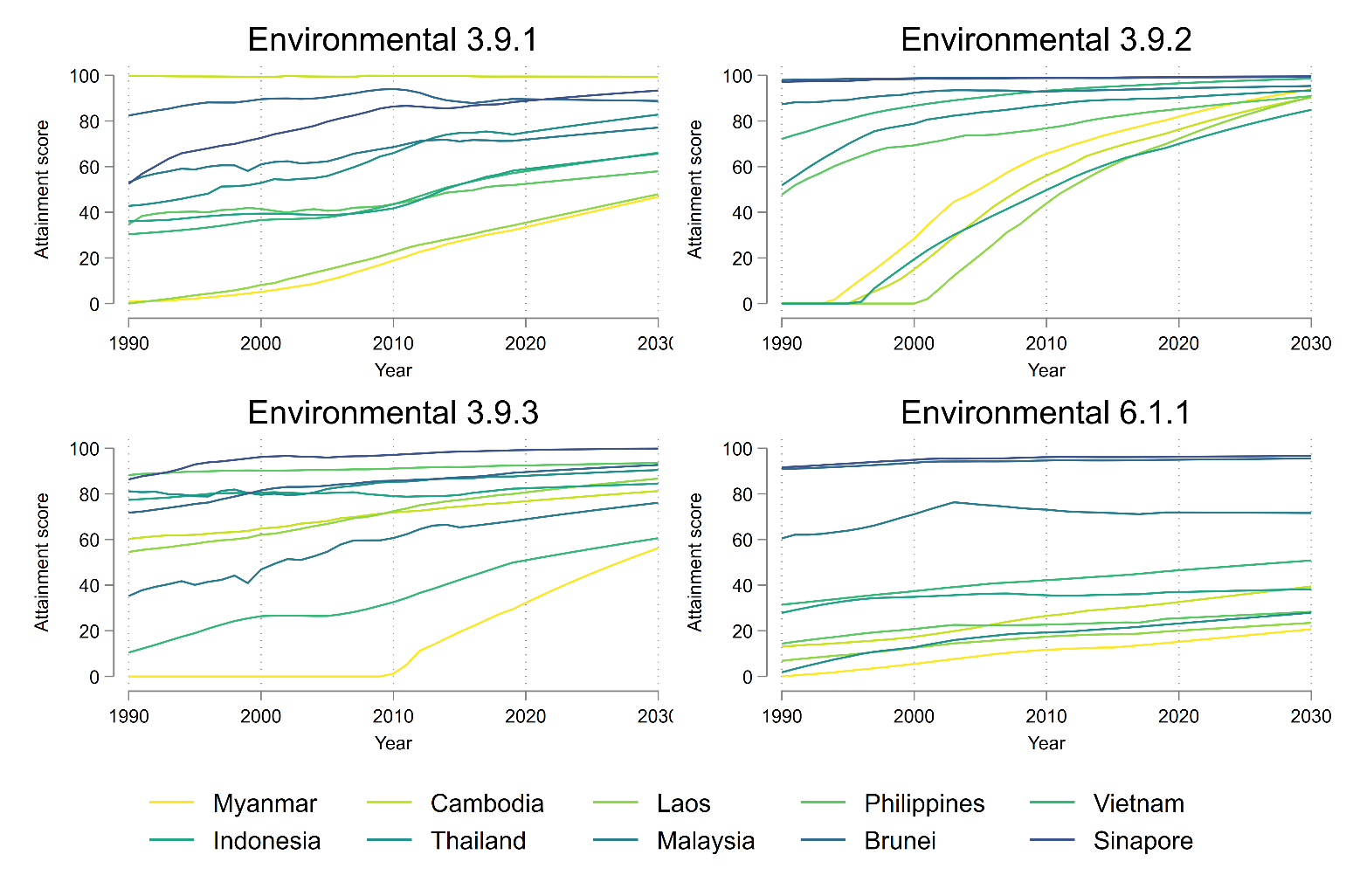


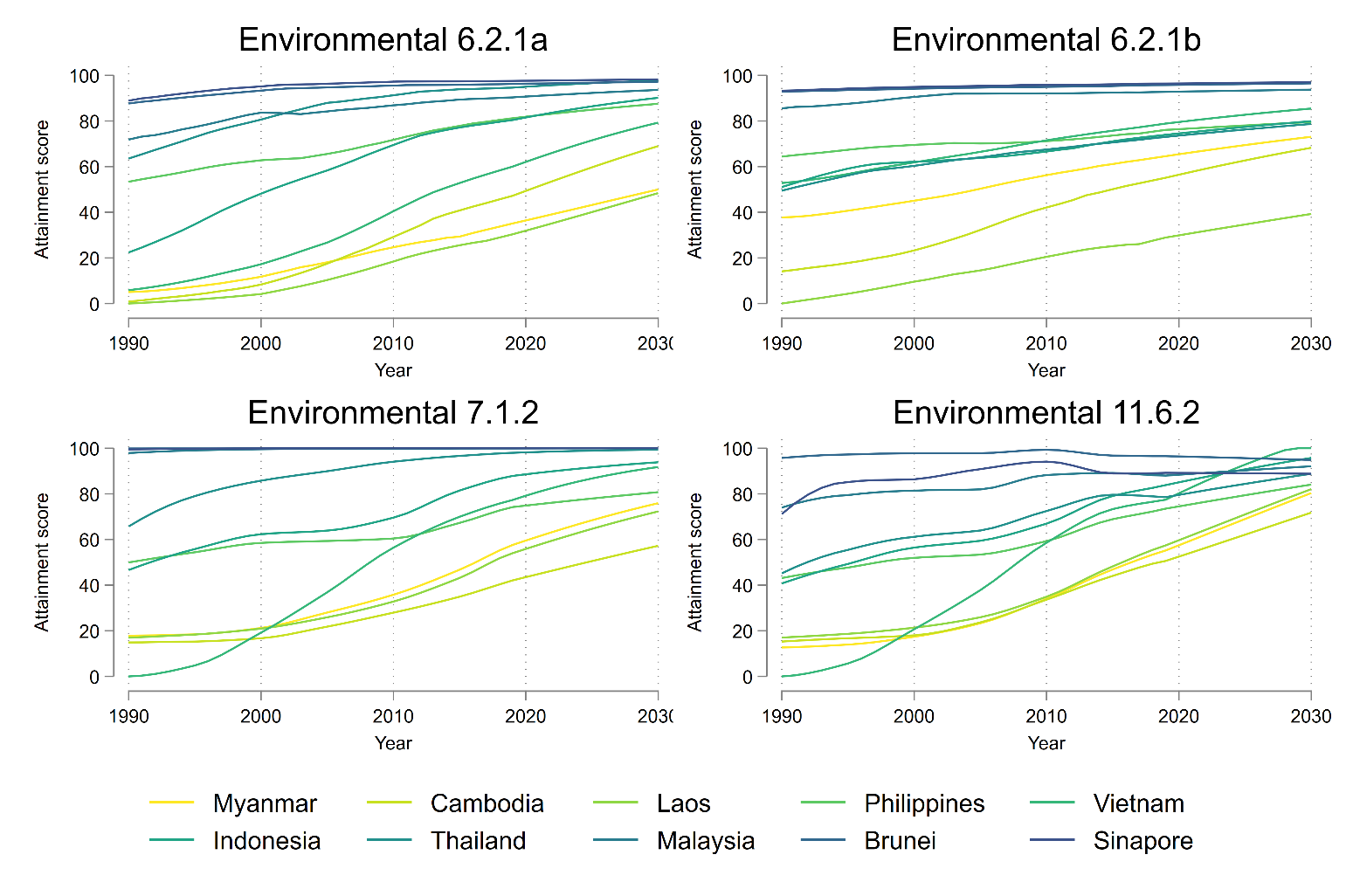


Note: In the legend, the countries are ranked in human development index in 2015, with darker blue indicating higher index.

## 1.6 Specific indicator for UHC


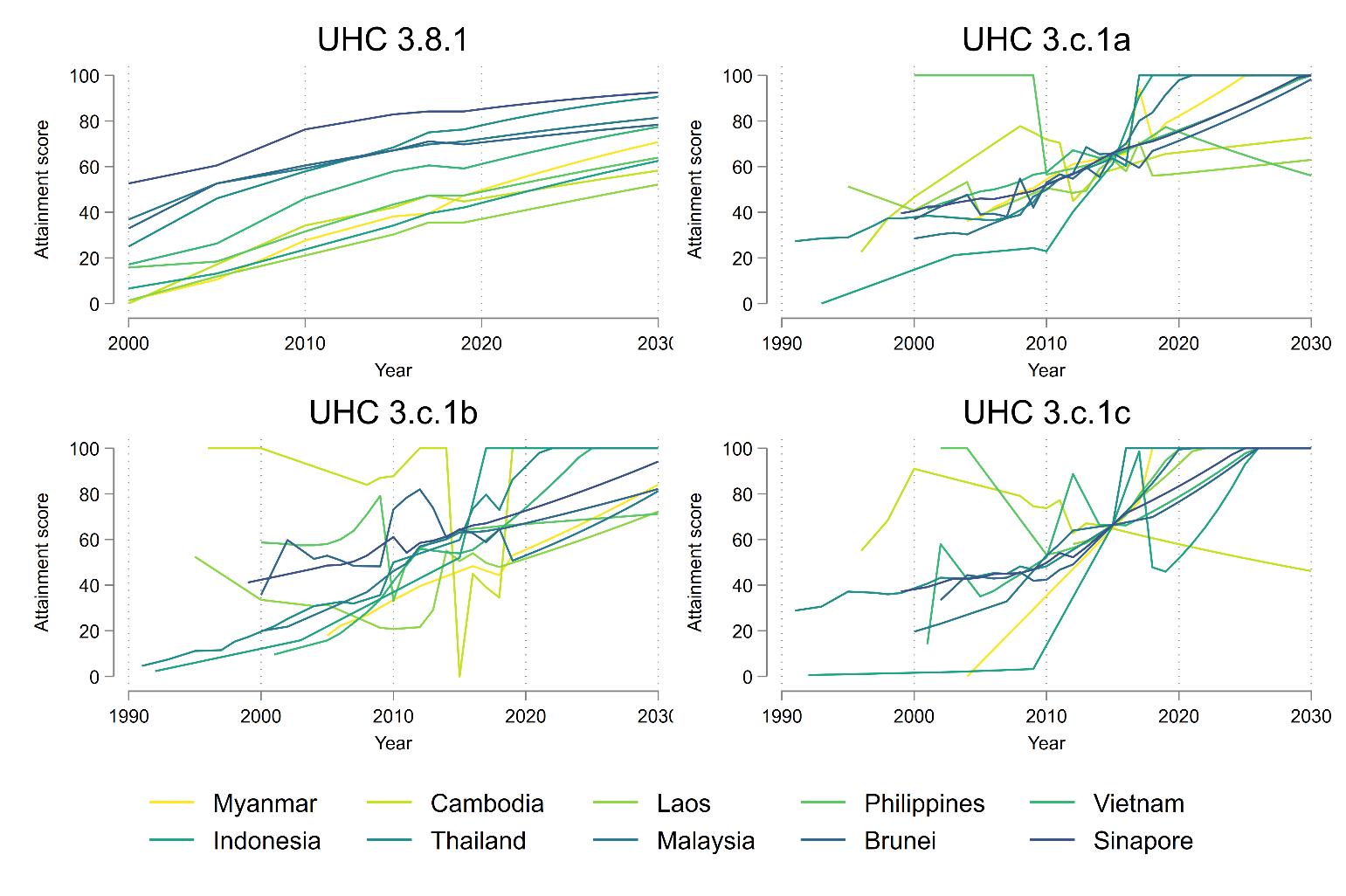


Note: In the legend, the countries are ranked in human development index in 2015, with darker blue indicating higher index.

## 1.7 Specific indicator in injury


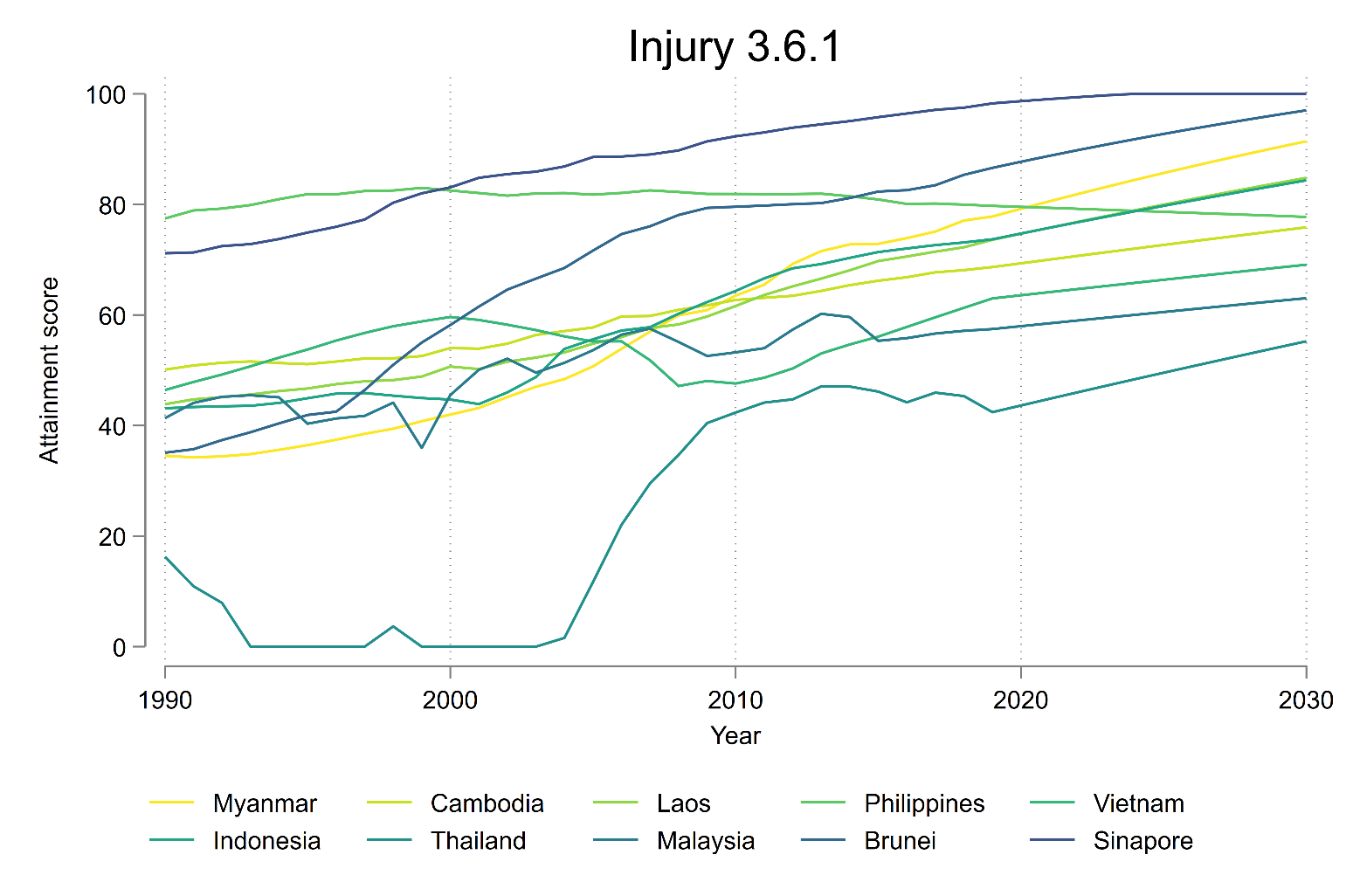


Note: In the legend, the countries are ranked in human development index in 2015, with darker blue indicating higher index.

# Figure A2 Regional disparities for specific SDG indicators

## 2.1 Specific indicators for nutrition


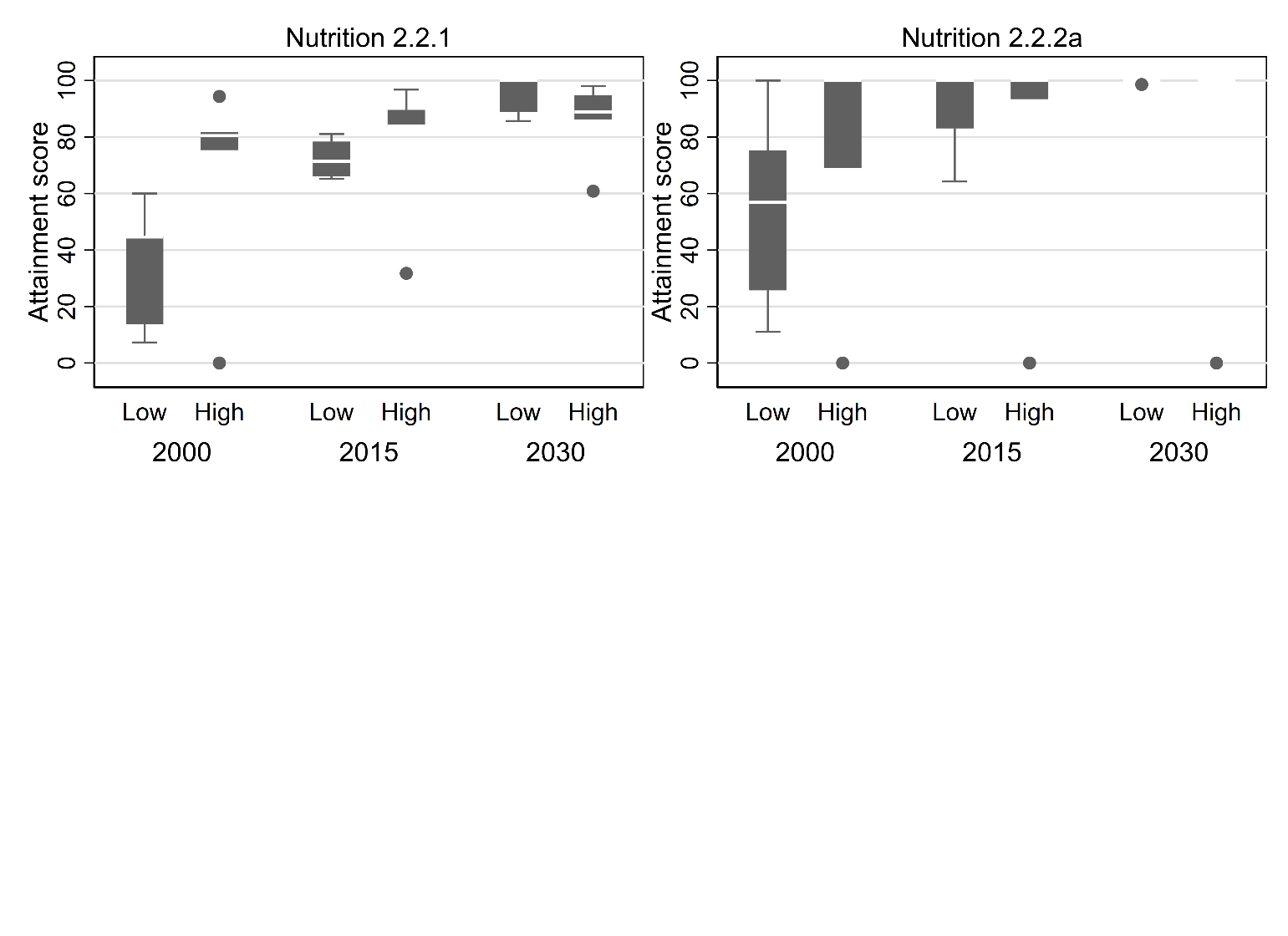


Note: HDI denotes human development index. Low HDI countries include Philippines, Indonesia, Vietnam, Laos, Cambodia, Myanmar using 0.800 as a cutoff. High HDI countries include Singapore, Brunei, Malaysia, Thailand. The center line inside the box represents the median. The box itself represents the interquartile range (IQR). Whiskers extend to the smallest and largest values within 1.5 × IQR from Q1 (25th percentile) and Q3 (75th percentile). Dots placed past the line edges to indicate outliers.

## 2.2 Specific indicators for MCH


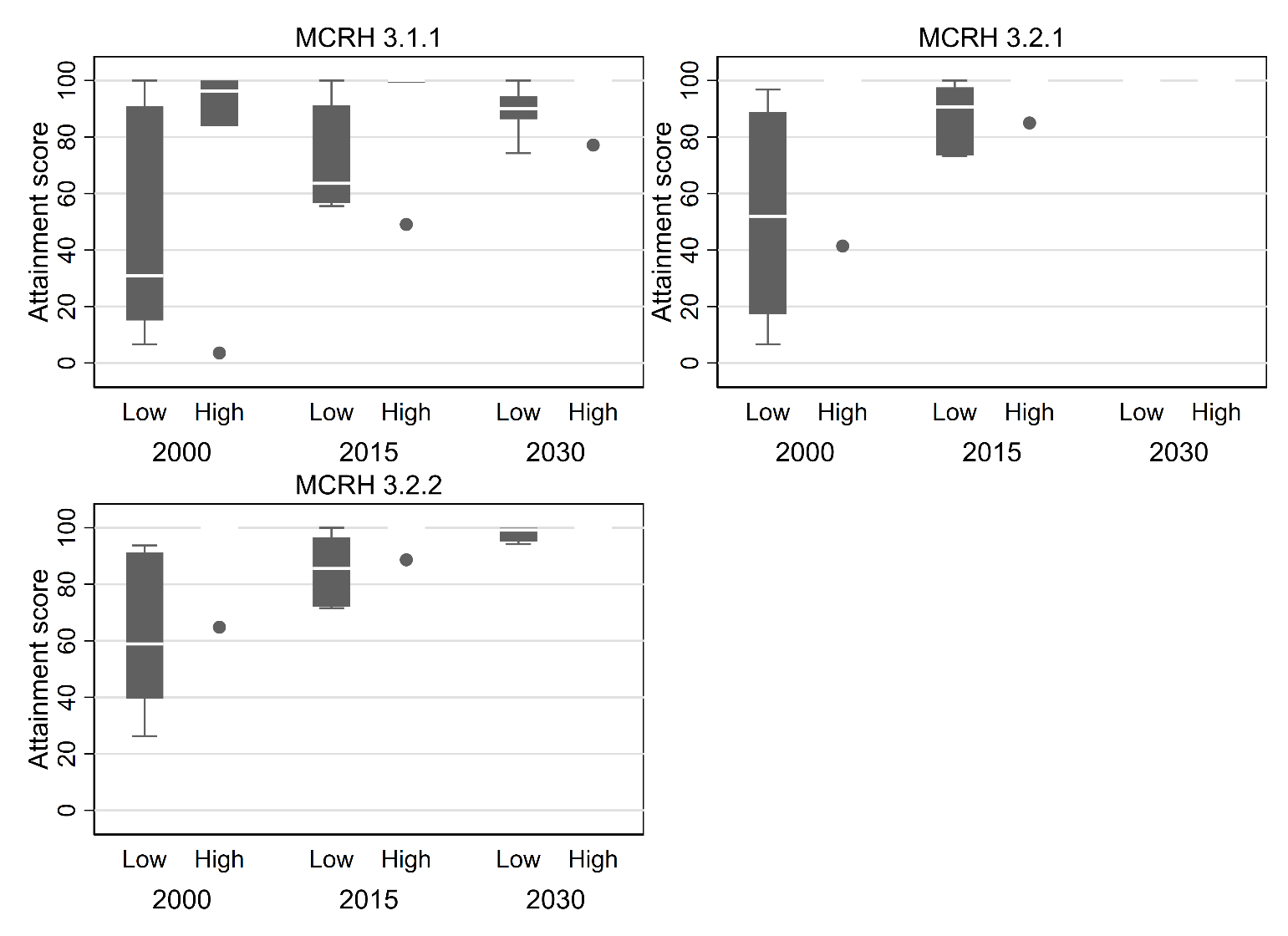


Note: HDI denotes human development index. Low HDI countries include Philippines, Indonesia, Vietnam, Laos, Cambodia, Myanmar using 0.800 as a cutoff. High HDI countries include Singapore, Brunei, Malaysia, Thailand. The center line inside the box represents the median. The box itself represents the interquartile range (IQR). Whiskers extend to the smallest and largest values within 1.5 × IQR from Q1 (25th percentile) and Q3 (75th percentile). Dots placed past the line edges to indicate outliers.

## 2.3 Specific indicators for infectious


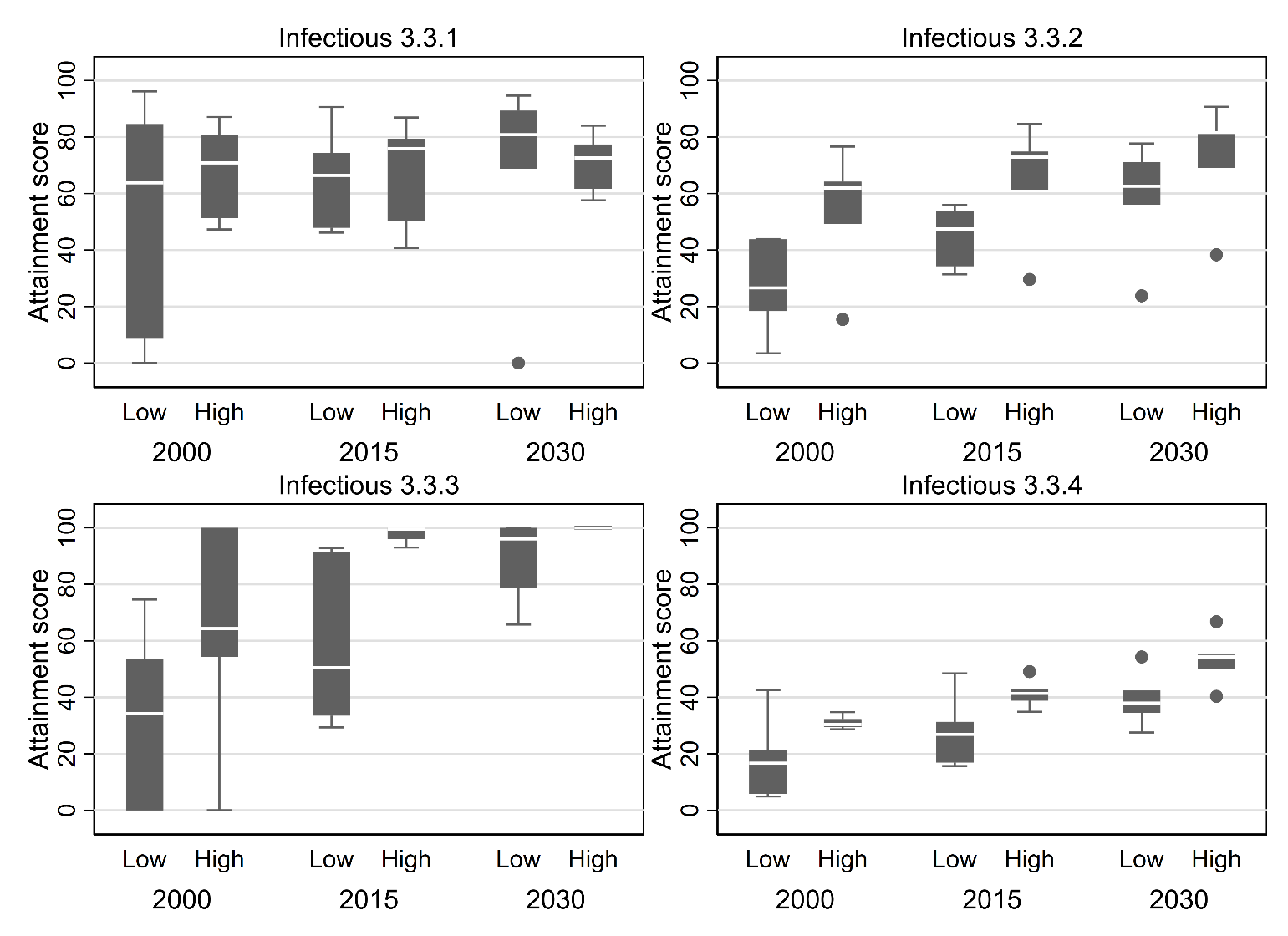


Note: HDI denotes human development index. Low HDI countries include Philippines, Indonesia, Vietnam, Laos, Cambodia, Myanmar using 0.800 as a cutoff. High HDI countries include Singapore, Brunei, Malaysia, Thailand. The center line inside the box represents the median. The box itself represents the interquartile range (IQR). Whiskers extend to the smallest and largest values within 1.5 × IQR from Q1 (25th percentile) and Q3 (75th percentile). Dots placed past the line edges to indicate outliers.


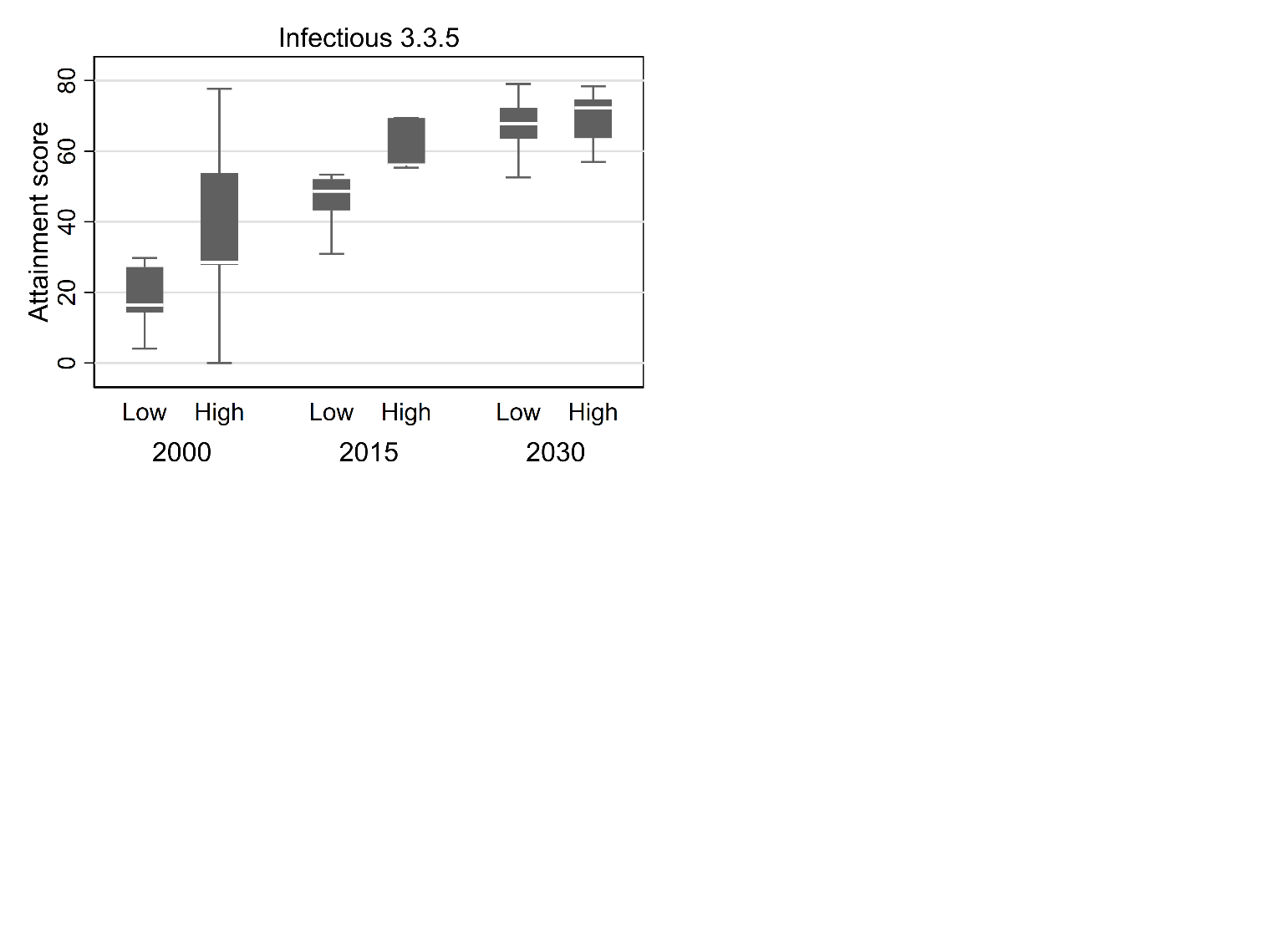


Note: HDI denotes human development index. Low HDI countries include Philippines, Indonesia, Vietnam, Laos, Cambodia, Myanmar using 0.800 as a cutoff. High HDI countries include Singapore, Brunei, Malaysia, Thailand. The center line inside the box represents the median. The box itself represents the interquartile range (IQR). Whiskers extend to the smallest and largest values within 1.5 × IQR from Q1 (25th percentile) and Q3 (75th percentile). Dots placed past the line edges to indicate outliers.

## 2.4 Specific indicators for NCD


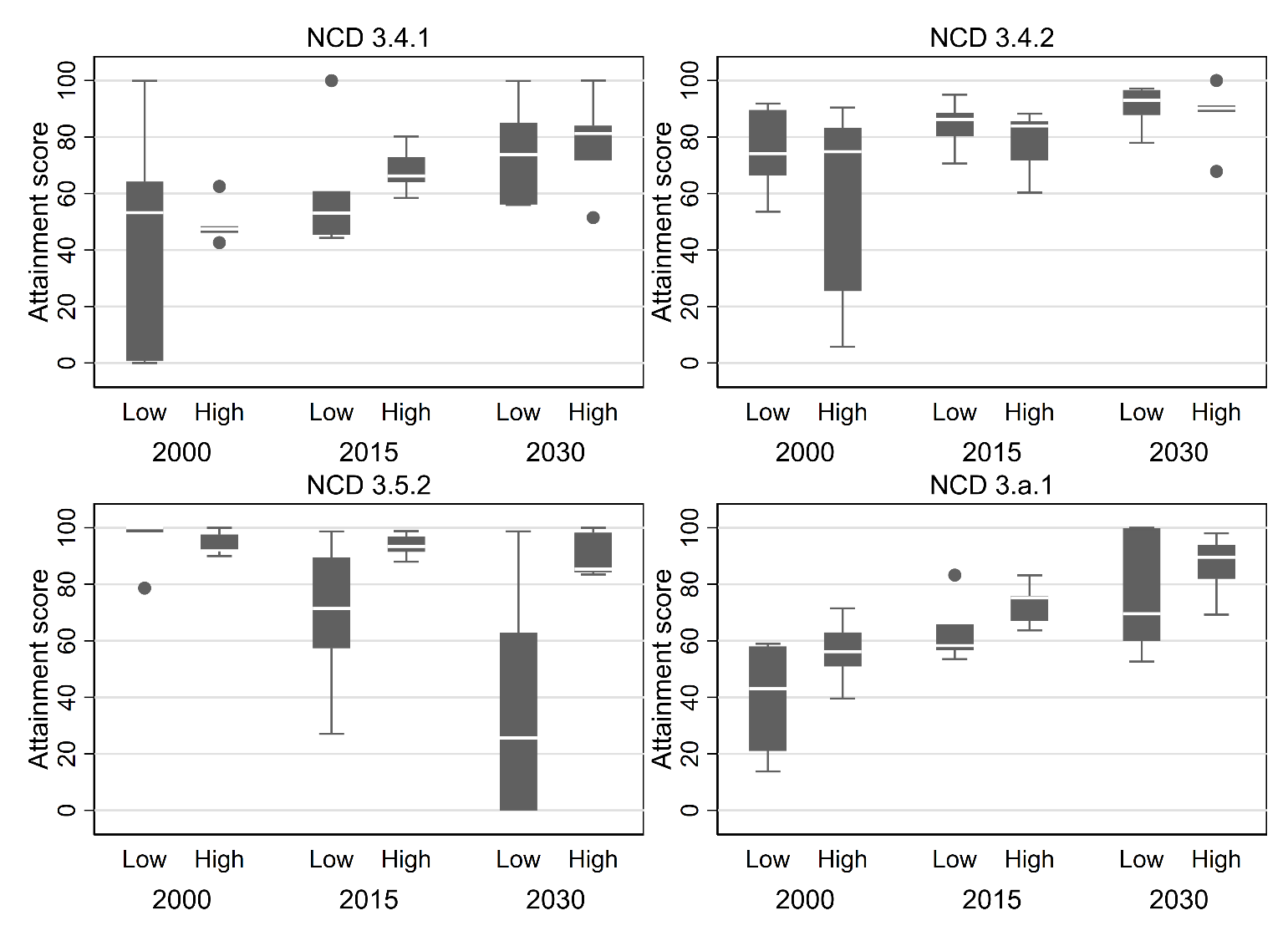


Note: HDI denotes human development index. Low HDI countries include Philippines, Indonesia, Vietnam, Laos, Cambodia, Myanmar using 0.800 as a cutoff. High HDI countries include Singapore, Brunei, Malaysia, Thailand. The center line inside the box represents the median. The box itself represents the interquartile range (IQR). Whiskers extend to the smallest and largest values within 1.5 × IQR from Q1 (25th percentile) and Q3 (75th percentile). Dots placed past the line edges to indicate outliers.

## 2.5 Specific indicators for environmental health


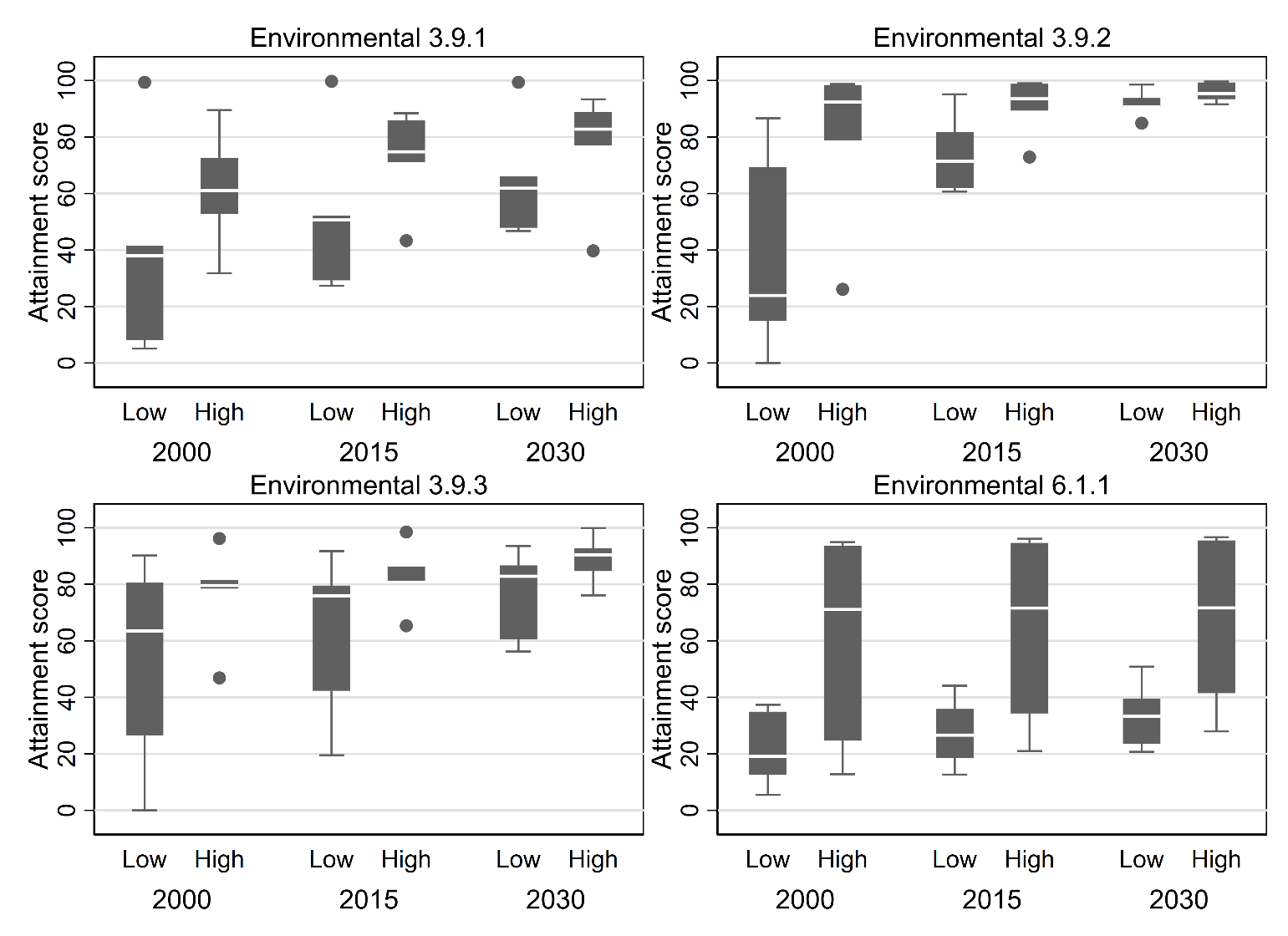


Note: HDI denotes human development index. Low HDI countries include Philippines, Indonesia, Vietnam, Laos, Cambodia, Myanmar using 0.800 as a cutoff. High HDI countries include Singapore, Brunei, Malaysia, Thailand. The center line inside the box represents the median. The box itself represents the interquartile range (IQR). Whiskers extend to the smallest and largest values within 1.5 × IQR from Q1 (25th percentile) and Q3 (75th percentile). Dots placed past the line edges to indicate outliers.


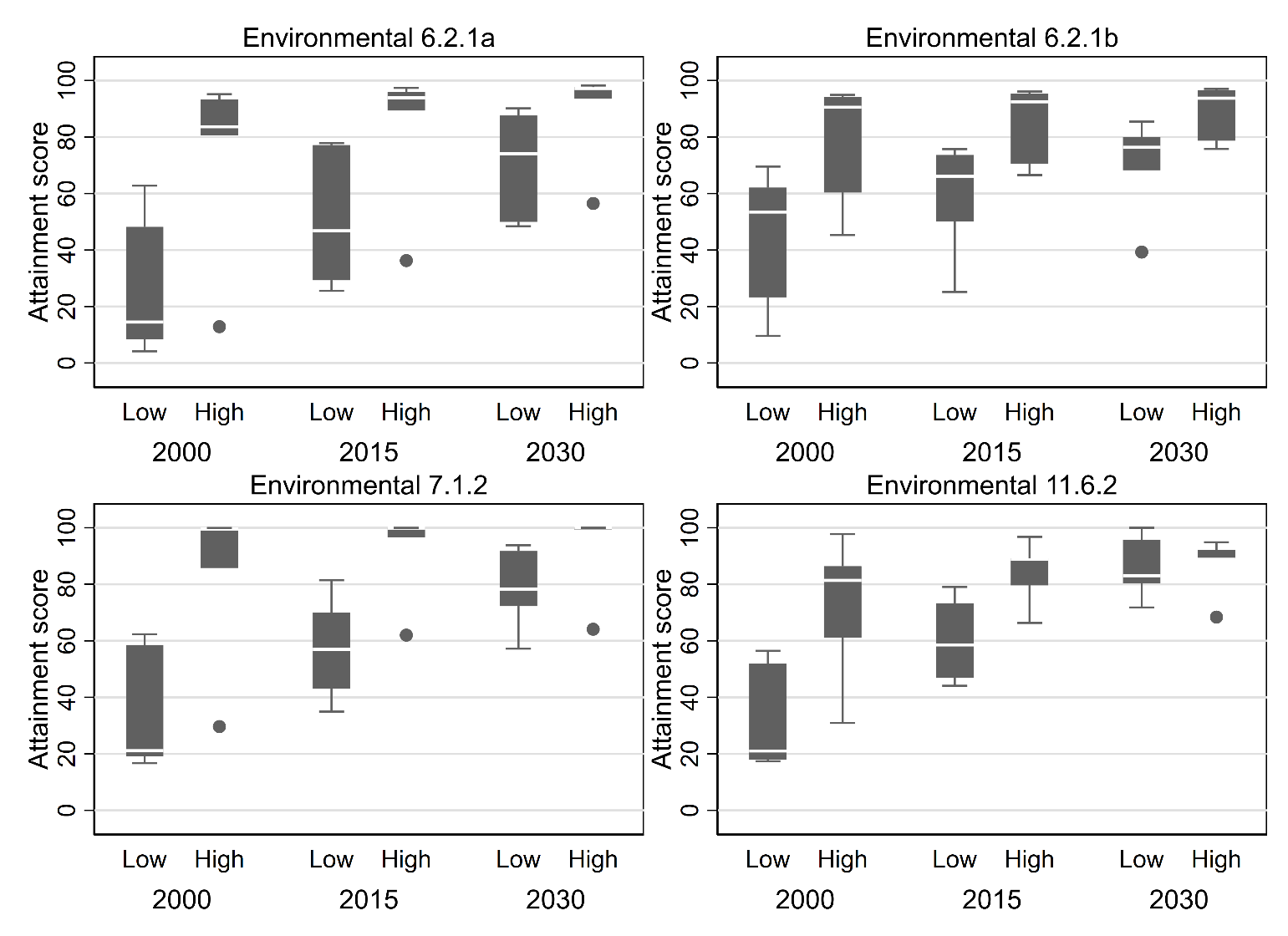


Note: HDI denotes human development index. Low HDI countries include Philippines, Indonesia, Vietnam, Laos, Cambodia, Myanmar using 0.800 as a cutoff. High HDI countries include Singapore, Brunei, Malaysia, Thailand. The center line inside the box represents the median. The box itself represents the interquartile range (IQR). Whiskers extend to the smallest and largest values within 1.5 × IQR from Q1 (25th percentile) and Q3 (75th percentile). Dots placed past the line edges to indicate outliers.

## 2.6 Specific indicators for UHC


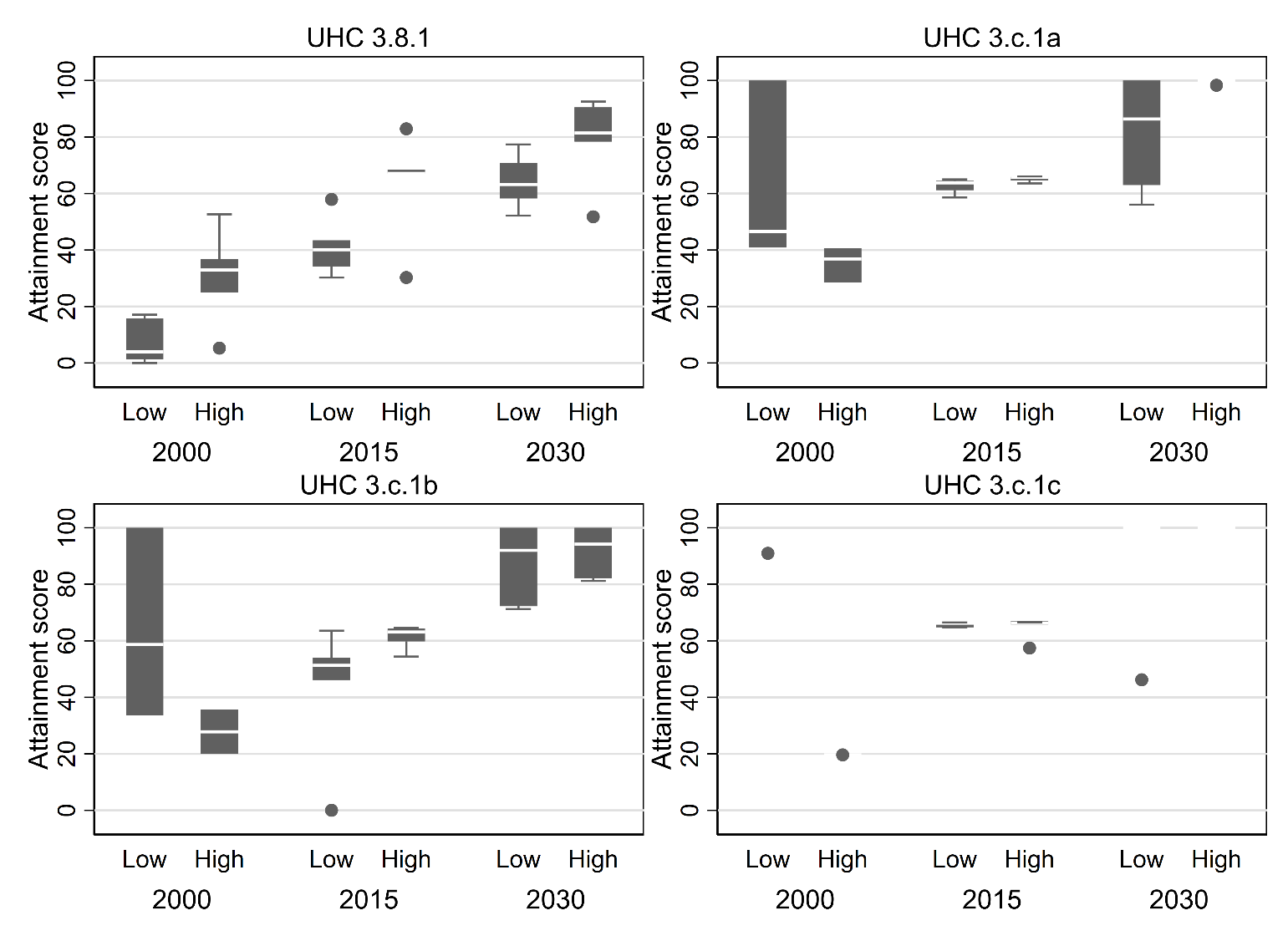


Note: HDI denotes human development index. Low HDI countries include Philippines, Indonesia, Vietnam, Laos, Cambodia, Myanmar using 0.800 as a cutoff. High HDI countries include Singapore, Brunei, Malaysia, Thailand. The center line inside the box represents the median. The box itself represents the interquartile range (IQR). Whiskers extend to the smallest and largest values within 1.5 × IQR from Q1 (25th percentile) and Q3 (75th percentile). Dots placed past the line edges to indicate outliers.

## 2.7 Specific indicators for injury


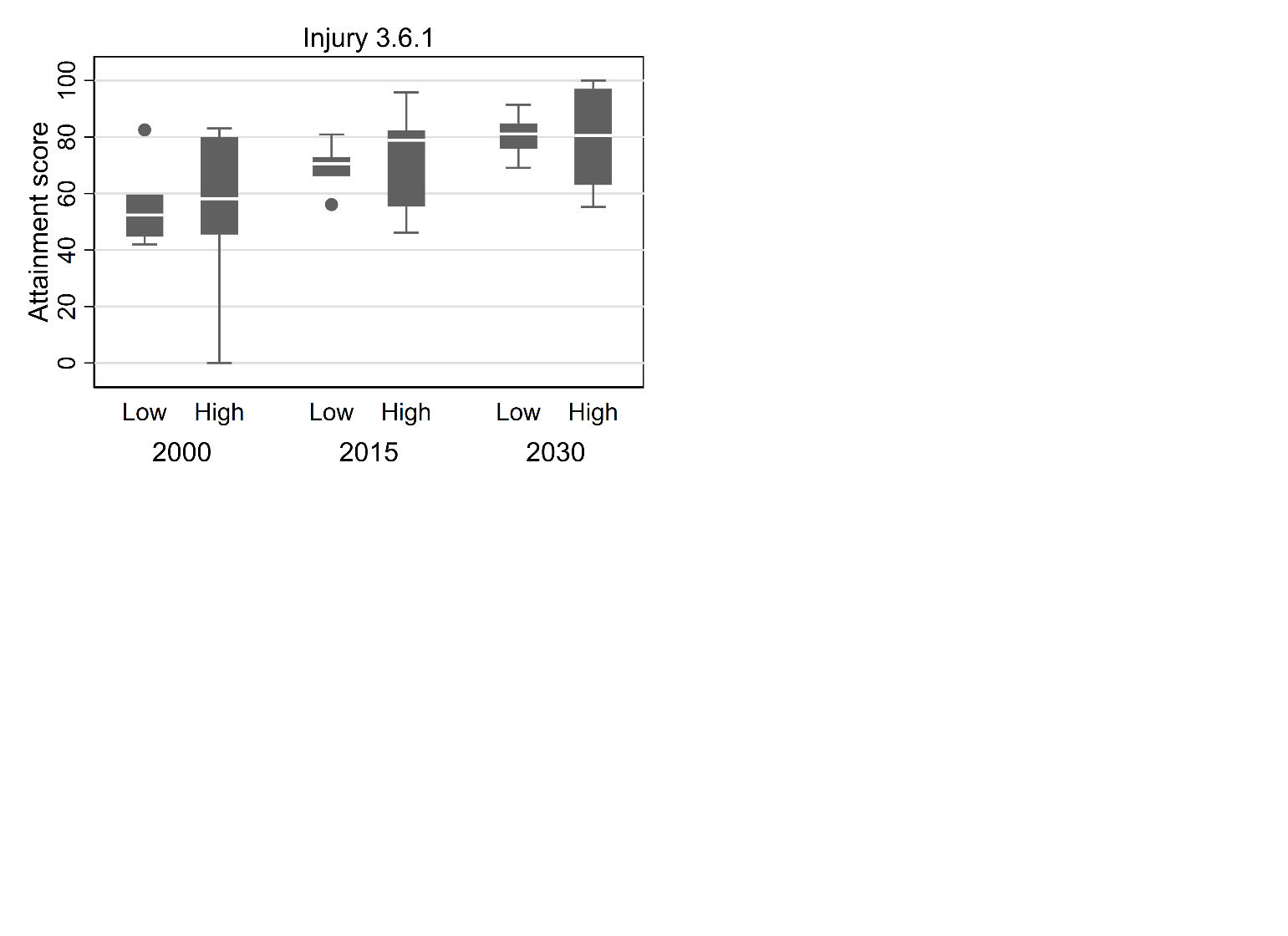


Note: HDI denotes human development index. Low HDI countries include Philippines, Indonesia, Vietnam, Laos, Cambodia, Myanmar using 0.800 as a cutoff. High HDI countries include Singapore, Brunei, Malaysia, Thailand. The center line inside the box represents the median. The box itself represents the interquartile range (IQR). Whiskers extend to the smallest and largest values within 1.5 × IQR from Q1 (25th percentile) and Q3 (75th percentile). Dots placed past the line edges to indicate outliers.

# Table A1 GBD 2021 estimates and uncertainty intervals in baseline (2015)

|  | **Brunei** | | | **Cambodia** | | | **Indonesia** | | | **Laos** | | | **Malaysia** | | | **Myanmar** | | | **Philippines** | | | **Singapore** | | | **Thailand** | | | **Viet Nam** | | |
| --- | --- | --- | --- | --- | --- | --- | --- | --- | --- | --- | --- | --- | --- | --- | --- | --- | --- | --- | --- | --- | --- | --- | --- | --- | --- | --- | --- | --- | --- | --- |
| **Outline** | **Value** | **Upper** | **Lower** | **Value** | **Upper** | **Lower** | **Value** | **Upper** | **Lower** | **Value** | **Upper** | **Lower** | **Value** | **Upper** | **Lower** | **Value** | **Upper** | **Lower** | **Value** | **Upper** | **Lower** | **Value** | **Upper** | **Lower** | **Value** | **Upper** | **Lower** | **Value** | **Upper** | **Lower** |
| 11.6.2 | 5.37 | 9.35 | 1.48 | 61.89 | 72.95 | 51.72 | 29.85 | 38.54 | 21.59 | 58.86 | 74.32 | 42.48 | 16.88 | 22.71 | 11.18 | 59.85 | 73.18 | 47.89 | 40.92 | 50.73 | 31.18 | 17.19 | 26.06 | 10.41 | 29.21 | 37.41 | 22.29 | 36.33 | 44.65 | 27.81 |
| 2.2.1 | 13.04 | 14.48 | 11.55 | 28.66 | 31.11 | 25.02 | 26.38 | 28.53 | 23.13 | 29.09 | 32.40 | 25.66 | 13.07 | 14.60 | 11.48 | 21.89 | 24.26 | 19.68 | 21.18 | 23.23 | 19.15 | 3.43 | 4.16 | 2.72 | 10.08 | 11.47 | 8.76 | 18.18 | 20.16 | 16.35 |
| 2.2.2a | 2.30 | 3.32 | 1.52 | 6.70 | 8.80 | 4.47 | 7.52 | 9.29 | 4.94 | 5.20 | 7.01 | 3.50 | 5.62 | 7.20 | 3.74 | 5.23 | 7.11 | 3.46 | 5.09 | 6.64 | 3.36 | 2.13 | 3.01 | 1.40 | 3.88 | 5.18 | 2.59 | 4.68 | 6.21 | 3.06 |
| 3.1.1 | 27.72 | 41.09 | 18.79 | 150.94 | 209.95 | 109.28 | 169.56 | 222.66 | 127.22 | 187.47 | 302.41 | 100.82 | 66.20 | 80.89 | 53.02 | 183.79 | 243.74 | 136.93 | 87.50 | 104.34 | 72.73 | 4.20 | 4.89 | 3.52 | 70.42 | 96.31 | 49.92 | 16.64 | 28.35 | 9.62 |
| 3.2.1 | 0.01 |  |  | 0.04 |  |  | 0.03 |  |  | 0.05 |  |  | 0.01 |  |  | 0.05 |  |  | 0.03 |  |  | 0.00 |  |  | 0.01 |  |  | 0.01 |  |  |
| 3.2.2 | 0.01 |  |  | 0.02 |  |  | 0.02 |  |  | 0.02 |  |  | 0.00 |  |  | 0.02 |  |  | 0.01 |  |  | 0.00 |  |  | 0.00 |  |  | 0.01 |  |  |
| 3.3.1 | 5.34 | 8.59 | 3.02 | 12.44 | 17.23 | 8.99 | 3.77 | 5.60 | 2.95 | 10.94 | 14.22 | 8.35 | 10.22 | 12.85 | 8.05 | 24.55 | 31.46 | 18.98 | 25.53 | 38.01 | 15.96 | 8.66 | 12.94 | 5.38 | 28.71 | 47.16 | 18.32 | 17.13 | 22.15 | 13.90 |
| 3.3.2 | 72.09 | 81.41 | 62.98 | 326.82 | 361.24 | 292.80 | 213.28 | 233.05 | 192.33 | 153.97 | 175.26 | 133.90 | 64.70 | 73.07 | 57.22 | 201.98 | 220.95 | 184.07 | 304.12 | 335.65 | 276.19 | 33.38 | 38.52 | 28.78 | 124.15 | 141.82 | 109.08 | 167.53 | 180.71 | 153.36 |
| 3.3.3 | 0.00 | 0.00 | 0.00 | 1283.87 | 1417.45 | 1149.00 | 288.91 | 315.42 | 265.24 | 1093.57 | 1257.32 | 934.59 | 1.17 | 1.42 | 0.96 | 821.09 | 930.41 | 720.69 | 26.43 | 31.39 | 22.01 | 0.00 | 0.00 | 0.00 | 20.22 | 21.93 | 18.71 | 21.18 | 23.06 | 19.51 |
| 3.3.4 | 492.35 | 653.88 | 356.80 | 1404.14 | 1929.99 | 957.80 | 1066.23 | 1305.00 | 873.95 | 1111.18 | 1518.15 | 805.04 | 661.96 | 784.76 | 556.12 | 507.14 | 633.54 | 407.09 | 1730.64 | 2212.18 | 1333.60 | 704.18 | 856.09 | 572.81 | 789.35 | 989.65 | 613.48 | 1795.60 | 2270.22 | 1383.60 |
| 3.3.5 | 524.80 | 2192.52 | 202.94 | 12880.21 | 18962.15 | 9040.02 | 12712.25 | 21729.10 | 7764.05 | 46312.57 | 55802.11 | 40280.50 | 8333.64 | 13904.82 | 5106.71 | 23466.07 | 29190.45 | 19038.72 | 12750.90 | 22638.51 | 8108.38 | 583.46 | 1244.93 | 158.19 | 10754.57 | 17694.80 | 7743.04 | 23142.43 | 36941.44 | 15369.33 |
| 3.4.1 | 3920.47 | 4546.68 | 3379.75 | 5112.00 | 6349.51 | 3978.11 | 5693.42 | 6658.94 | 4770.21 | 6263.74 | 7963.29 | 4798.70 | 4217.62 | 4698.71 | 3762.14 | 6176.80 | 7759.39 | 4801.31 | 5233.53 | 5623.49 | 4885.02 | 1733.21 | 1821.76 | 1637.73 | 2943.84 | 3468.79 | 2480.54 | 4612.36 | 5590.14 | 3664.33 |
| 3.4.2 | 3.96 | 4.53 | 3.24 | 5.08 | 6.42 | 3.89 | 1.84 | 2.18 | 1.57 | 5.99 | 8.06 | 4.56 | 5.12 | 5.74 | 4.73 | 3.87 | 4.79 | 2.55 | 3.91 | 4.23 | 3.50 | 7.79 | 8.12 | 7.41 | 9.66 | 11.02 | 8.36 | 7.99 | 9.89 | 6.15 |
| 3.5.2 | 2.03 | 4.96 | 0.93 | 11.30 | 19.19 | 7.67 | 2.15 | 4.60 | 1.13 | 17.15 | 25.68 | 11.28 | 4.63 | 11.40 | 2.64 | 5.13 | 10.03 | 3.37 | 17.52 | 26.44 | 12.97 | 8.44 | 15.89 | 5.69 | 12.70 | 20.27 | 9.67 | 11.07 | 17.75 | 7.48 |
| 3.6.1 | 10.92 | 12.13 | 9.83 | 19.74 | 24.76 | 15.80 | 17.05 | 19.86 | 14.70 | 17.91 | 22.63 | 14.16 | 24.95 | 26.44 | 23.63 | 16.25 | 19.55 | 13.40 | 11.76 | 12.56 | 10.98 | 2.69 | 2.79 | 2.57 | 28.90 | 32.30 | 25.80 | 24.60 | 29.48 | 20.08 |
| 3.8.1 | 0.75 |  |  | 0.56 |  |  | 0.50 |  |  | 0.47 |  |  | 0.75 |  |  | 0.53 |  |  | 0.57 |  |  | 0.87 |  |  | 0.76 |  |  | 0.68 |  |  |
| 3.9.1 | 20.51 | 34.13 | 6.76 | 238.34 | 290.85 | 183.18 | 139.51 | 183.58 | 100.58 | 260.23 | 329.32 | 198.33 | 65.65 | 87.98 | 45.40 | 271.35 | 331.48 | 220.04 | 153.44 | 189.98 | 115.71 | 25.80 | 38.71 | 14.67 | 54.30 | 69.63 | 40.26 | 139.10 | 167.42 | 106.36 |
| 3.9.2 | 0.85 | 2.22 | 0.02 | 26.42 | 51.71 | 7.12 | 32.57 | 50.99 | 14.07 | 33.81 | 55.27 | 13.98 | 4.77 | 9.42 | 1.52 | 20.51 | 32.10 | 10.83 | 14.21 | 25.73 | 5.63 | 0.70 | 2.02 | -0.09 | 8.03 | 13.74 | 3.31 | 3.58 | 7.28 | 0.77 |
| 3.9.3 | 0.25 | 0.32 | 0.21 | 0.49 | 0.74 | 0.34 | 0.39 | 0.54 | 0.15 | 0.43 | 0.67 | 0.30 | 0.65 | 0.87 | 0.56 | 1.37 | 1.86 | 0.59 | 0.16 | 0.19 | 0.13 | 0.03 | 0.03 | 0.03 | 0.26 | 0.31 | 0.19 | 1.03 | 1.41 | 0.39 |
| 3.a.1 | 16.77 | 18.89 | 14.89 | 19.86 | 21.49 | 18.43 | 18.16 | 18.96 | 17.41 | 18.08 | 19.54 | 16.68 | 15.86 | 16.75 | 15.08 | 12.89 | 13.80 | 12.07 | 19.51 | 20.35 | 18.64 | 12.08 | 13.01 | 11.05 | 14.63 | 15.28 | 13.94 | 18.29 | 19.16 | 17.50 |
| 3.c.1a | 17.54 |  |  | 1.98 |  |  | 2.73 |  |  | 4.00 |  |  | 14.96 |  |  | 6.23 |  |  | 6.22 |  |  | 22.05 |  |  | 4.55 |  |  | 8.01 |  |  |
| 3.c.1b | 65.40 |  |  | 5.93 |  |  | 12.96 |  |  | 12.12 |  |  | 40.76 |  |  | 10.36 |  |  | 46.28 |  |  | 59.48 |  |  | 23.15 |  |  | 14.30 |  |  |
| 3.c.1c | 1.69 |  |  | 0.34 |  |  | 1.17 |  |  | 2.09 |  |  | 3.38 |  |  | 0.34 |  |  | 3.27 |  |  | 4.69 |  |  | 1.74 |  |  | 3.42 |  |  |
| 6.1.1 | 3.84 | 7.57 | 1.77 | 50.59 | 75.73 | 30.19 | 46.17 | 72.90 | 22.56 | 58.67 | 87.19 | 35.49 | 20.44 | 32.97 | 10.19 | 62.87 | 97.49 | 36.29 | 55.02 | 82.46 | 32.23 | 2.80 | 4.95 | 1.32 | 56.88 | 87.40 | 31.68 | 40.24 | 68.22 | 16.99 |
| 6.2.1a | 3.67 | 5.36 | 2.34 | 52.69 | 59.09 | 45.94 | 20.35 | 25.24 | 15.94 | 66.26 | 74.05 | 57.39 | 9.44 | 13.13 | 6.40 | 62.88 | 72.30 | 54.81 | 19.70 | 26.30 | 14.34 | 2.31 | 3.43 | 1.45 | 5.43 | 8.13 | 3.49 | 42.00 | 51.12 | 33.38 |
| 6.2.1b | 2.49 | 4.04 | 0.97 | 26.61 | 38.79 | 11.11 | 15.41 | 23.14 | 5.86 | 39.93 | 54.62 | 16.89 | 4.01 | 6.78 | 1.42 | 20.74 | 31.43 | 8.04 | 14.07 | 22.97 | 5.82 | 2.08 | 3.56 | 0.74 | 15.69 | 23.91 | 6.30 | 12.95 | 20.74 | 5.11 |
| 7.1.2 | 0.02 | 0.01 | 0.00 | 51.49 | 64.75 | 39.20 | 14.67 | 27.02 | 6.25 | 44.98 | 67.19 | 25.35 | 0.08 | 0.71 | 0.00 | 42.14 | 61.27 | 24.93 | 25.89 | 39.81 | 14.18 | 0.01 | 0.01 | 0.00 | 2.66 | 14.15 | 0.09 | 23.77 | 37.13 | 12.38 |

Note: Data was extracted from GBD 2021 estimates.
